# A Concise Asymmetric Synthesis of Sex Pheromone of *Euproctis pseudoconspersa* (Strand) and Its Enantiomer

**DOI:** 10.3390/molecules30122494

**Published:** 2025-06-06

**Authors:** Biyu An, Shengli Liu, Jianan Wang, Dan Liu, Qinghua Bian, Jiangchun Zhong

**Affiliations:** Department of Applied Chemistry, China Agricultural University, Beijing 100193, China; aby19990129@163.com (B.A.); xxjnwang@163.com (J.W.);

**Keywords:** tea tussock moth, chiral auxiliary, sex pheromone, asymmetric synthesis

## Abstract

The tea tussock moth, *Euproctis pseudoconspersa* (Strand), is a serious pest, and its sex pheromone is (*R*)-10,14-dimethylpentadecyl isobutyrate. A new and concise asymmetric synthesis of the sex pheromone and its enantiomer was accomplished. The chiral methyl of the pheromone was introduced by Evans’s template, while the extension of the carbon chain was achieved through Li_2_CuCl_4_-catalyzed coupling of chiral tosylate with Grignard reagent.

## 1. Introduction

The tea tussock moth, *Euproctis pseudoconspersa* (Strand) (Lepidoptera: Lymantridae), is a serious pest found in the tea plant, *Camellia sinensis*; the Japanese camellia, *Camellia japonica*; and the tea-oil plant, *Camellia oleifera* [1,2]. This pest is distributed throughout China, Korea, and Japan [3,4]. The larva feed on tea leaves and have caused significant damage to tea plantations [5]. Moreover, the venomous hair on the larvae, pupae, and adults could cause irritation and inflammation of human skin [6,7]. The conventional chemical insecticides have disadvantages of toxicity to mammals, negative environmental impacts, and inducing pest resistance; it would be meaningful to search for a more environment-friendly and effective alternative to control the tea tussock moth [8,9].

**Scheme 1:**
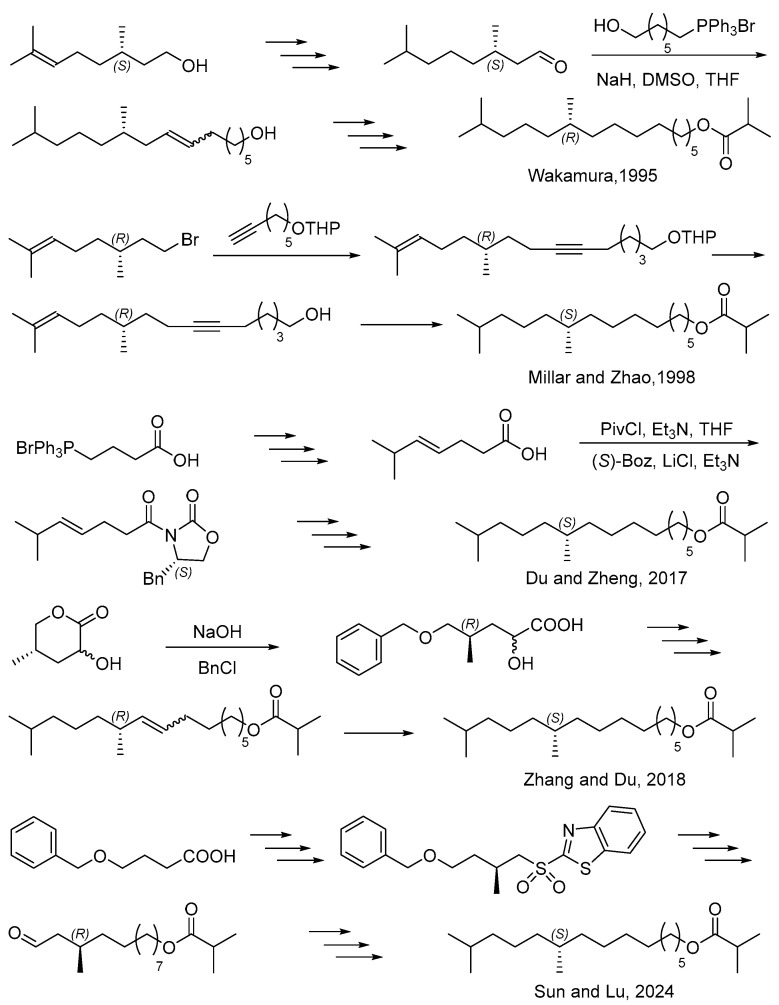
Previous syntheses of sex pheromone of *E. pseudoconspersa* [13,14,17,18,19].

Pheromone-based pest control is an alternative strategy with the advantages of being environment benign, effective, and rarely inducing pest resistance [10,11]. In 1994, Wakamura identified the sex pheromone of *E. pseudoconspersa* as 10,14-dimethylpentadecyl isobutyrate [12]. Later, Wakamura synthesized (*R*)- and (*S*)-10,14-dimethylpentadecyl isobutyrate from (*S*)- and (*R*)-citronellol, and also found that the *R* enantiomer was as active as the natural pheromone [13]. The sex pheromone could attract the male tea tussock moth [14,15]. Therefore, it could be used for mass trapping and significantly reduce mating rates [16]; this motivated the synthesis of the sex pheromone. In 1998, Millar and Zhao developed a concise synthesis of the sex pheromone from the chiral source of (*R*)-(−)-citronellyl bromide [14]. In 2017, Du and Zheng completed the asymmetric synthesis of the pheromone by Evans’s template and Julia–Kocienski coupling [17]. In 2018, they developed another synthesis from the recycling of wastewater from the steroid industry [18]. Recently, they developed a novel synthesis of the sex pheromone based on Evans’s template [19] (Scheme 1).

**Figure 1:**
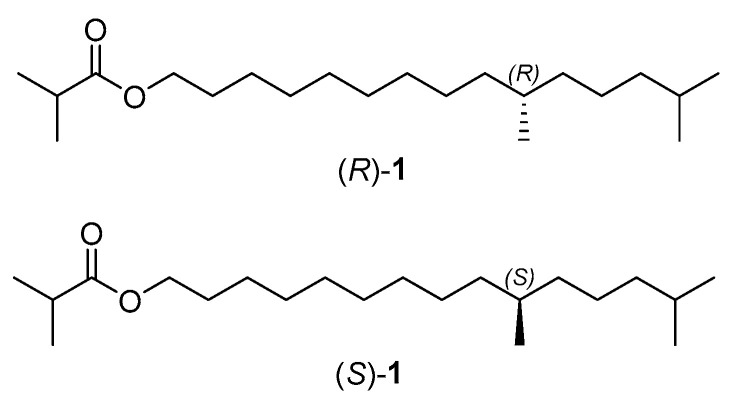
Sex pheromone of *E. pseudoconspersa* and its enantiomer.

To research further biological activities of the two enantiomers, herein a new and concise synthesis of the sex pheromone of the tea tussock moth and its enantiomer (Figure 1) has been achieved.

## 2. Results and Discussion

### 2.1. Retrosynthetic Analysis

**Scheme 2:**
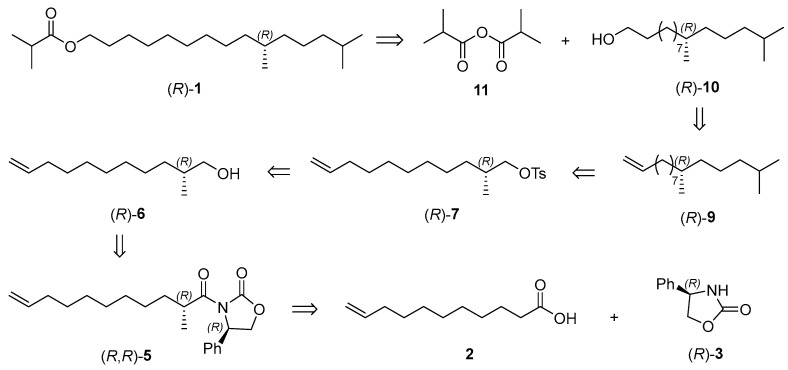
Retrosynthetic analysis of sex pheromone of *E. pseudoconspersa* (*R*)-**1**.

The retrosynthetic analysis of the sex pheromone of *E. pseudoconspersa*, (*R*)-10,14-dimethylpentadecyl isobutyrate ((*R*)-**1**) is illustrated in Scheme 2. The target pheromone (*R*)-**1** could be prepared through an esterification of (*R*)-10,14-dimethylpentadecan-1-ol ((*R*)-**10**) and isobutyric anhydride (**11**). Chiral alcohol (*R*)-**10** could be prepared by Li_2_CuCl_4_ catalyzed coupling of chiral tosylate (*R*)-**7** with a Grignard reagent. Evans’s chiral auxiliaries have been used in the synthesis of many natural products, which could induce chirality with high enantiomerical purity [20,21]. Therefore, we envisaged that this strategy could construct the stereocenter of chiral alcohol (*R*)-**6**, and the starting materials could be unsaturated acid **2** and (*R*)-4-phenyl-2-oxazolidinone ((*R*)-**3**). Following the similar procedure for sex pheromone (*R*)-**1**, its enantiomer (*S*)-10,14-dimethylpentadecyl isobutyrate ((*S*)-**1**) could be prepared.

### 2.2. Synthesis of Chiral Alcohols (R)- and (S)-***6***

**Scheme 3:**
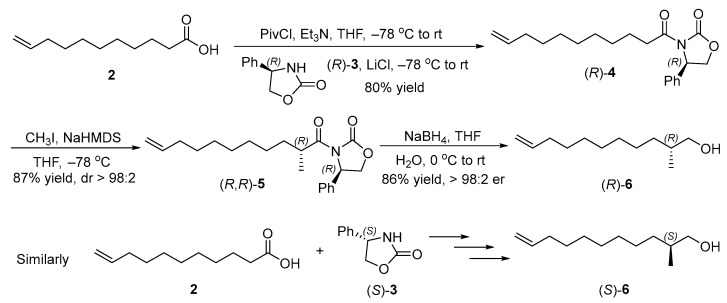
Synthesis of chiral alcohols (*R*)- and (*S*)-**6**.

According to the retrosynthetic analysis of the sex pheromone (*R*)-**1**, our synthesis began with the preparation of chiral alcohols (*R*)- and (*S*)-**6** (Scheme 3). The reaction of undec-10-enoic acid (**2**) with pivaloyl chloride provided the corresponding mixed anhydride, followed by the acylation with (*R*)-4-phenyloxazolinone ((*R*)-**3**) to afford imide (*R*)-**4** [22,23]. The chiral methyl of imide (*R*,*R*)-**5** was introduced by the diastereoselective methylation with CH_3_I [24], and the absolute configuration of the new stereocenter was assigned as (*R*) according to Evans’s auxiliary precedent [25]. The dr was more than 98:2, determined by ^13^C NMR spectra of imide (*R*,*R*)-**5**. The final treatment with NaBH_4_ resulted in the formation of (*R*)-2-methylpentadec-14-en-1-ol ((*R*)-**6**) (86% yield, >98:2 er, based on the signal/noise ratio of the ^19^F NMR spectra of its Mosher ester) [26]. Its specific rotations were consistent with the literature value [27], which supported (*R*)-configuration of chiral alcohol (*R*)-**6**. Similarly, (*S*)-2-methylundec-10-en-1-ol ((*S*)-**6**) (>98:2 er, based on the signal/noise ratio of the ^19^F NMR spectra of its Mosher ester) was prepared via the acylation of unsaturated acid **2** with (*S*)-4-phenyl-2-oxazolidinone ((*S*)-**3**), diastereoselective methylation of imide (*S*)-**4**, and the reductive cleavage of the chiral auxiliary from imide (*S*,*S*)-**5** with NaBH_4_.

### 2.3. Synthesis of Chiral Alcohols (R)- and (S)-***10***

**Scheme 4:**
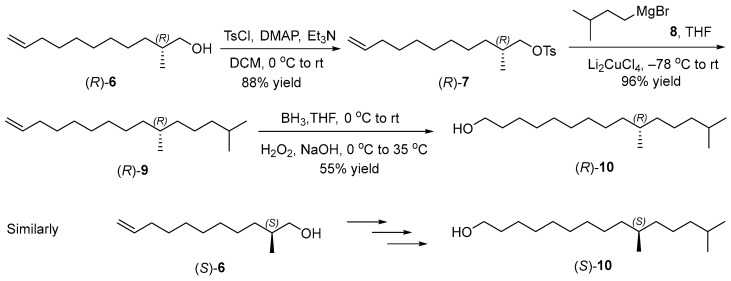
Synthesis of chiral alcohols (*R*)- and (*S*)-**10**.

Having completed the synthesis of the chiral alcohols (*R*)- and (*S*)-**6**, we next prepared chiral alcohols (*R*)- and (*S*)-**10** (Scheme 4). Chiral alcohol (*R*)-**6** was treated with TsCl to afford (*R*)-2-methylpentadec-14-en-1-yl 4-methylbenzenesulfonate ((*R*)**-7**) in 88% yield [28], which was converted to (*R*)-10,14-dimethylpentadec-1-ene ((*R*)-**9**) smoothly through the reaction with isopentyl magnesium bromide (**8**) in the presence of Li_2_CuCl_4_ [29]. The subsequent hydroboration-oxidation of (*R*)-10,14-dimethylpentadec-1-ene ((*R*)-**9**) provided (*R*)-10,14-dimethylpentadecan-1-ol ((*R*)-**10**) [30]. Following the similar procedure for chiral alcohol (*R*)-**10**, (*S*)-10,14-dimethylpentadecan-1-ol ((*S*)-**10**) was synthesized by the tosylation of chiral alcohol (*S*)-**6**, Li_2_CuCl_4_-catalyzed coupling of chiral tosylate (*S*)-**7** with Grignard reagent and hydroboration-oxidation of terminal olefin (*S*)-**9**.

### 2.4. Synthesis of Sex Pheromone of E. pseudoconspersa and Its Enantiomer

**Scheme 5:**
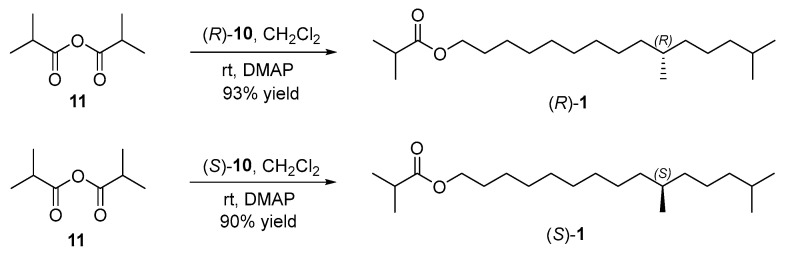
Synthesis of sex pheromone of *E. pseudoconspersa* and its enantiomer.

With the key chiral alcohols (*R*)- and (*S*)-**10** in hand, the sex pheromone of *E. pseudoconspersa* and its enantiomer were synthesized (Scheme 5). In the presence of 4-(dimethylamino)pyridine, the reaction of isobutyric anhydride (**11**) with chiral alcohol (*R*)-**10** generated the target pheromone (*R*)-**1** in 93% yield [31]. Its enantiomer (*S*)-10,14-dimethylpentadecyl isobutyrate ((*S*)-**1**) was also prepared by the esterification of chiral alcohol (*S*)-**10** with isobutyric anhydride (**11**). Their NMR spectra, HRMS, and the specific rotation were consistent with the references [18,19].

## 3. Materials and Methods

### 3.1. General Information

Unless otherwise stated, all reactions were conducted in oven-dried glassware with a Schlenk line under an argon atmosphere, and all reaction mixtures were magnetically stirred. Tetrahydrofuran, triethylamine, and diethyl ether were distilled from CaH_2_ before use, and all starting materials and reagents were purchased from commercial sources. Optical rotations were determined by a Rudolph AUTOPOL-IV polarimeter (Rudolph Research Analytical, Hackettstown, NJ, USA), with a 0.25 dm cell length. ^1^H and ^13^C NMR spectra were recorded on a Bruker AscendTM 500 MHz spectrometer (Bruker, Billerica, MA, USA) and using deuterated solvent. Chemical shifts were reported in parts per million (ppm) with an internal standard of tetramethylsilane (0.00 ppm) for ^1^H NMR and the residual protium CDCl_3_ (77.16 ppm) for ^13^C NMR spectra. High-resolution mass spectra (HRMS) analyses were performed on Waters LCT Premier™ with an ESI mass spectrometer (Waters, Milford, MA, USA).

### 3.2. Synthesis of (R)-4-Phenyl-3-(undec-10-enoyl)oxazolidin-2-one ((R)-***4***) (New Compound)

To a 500 mL three-neck flask were added undec-10-enoic acid (**2**) (5.00 g, 27.13 mmol) Et_3_N (5.77 g, 56.97 mmol) and THF (150 mL) at room temperature. The solution was cooled to −78 °C, then pivaloyl chloride (3.93 g, 32.55 mmol) was added and stirred for 0.5 h. The reaction solution was allowed to warm to room temperature and stirred for 1 h. (*R*)-4-Phenyl-2-oxazolidinone ((*R*)-**3**) (4.87 g, 29.84 mmol) in THF (40 mL) and LiCl (3.45 g, 81.39 mmol) were then added. The reaction mixture was cooled to −78 °C again and stirred for 1 h. After the reaction mixture was allowed to warm to room temperature and stirred for additional 10 h, it was quenched with saturated NH_4_Cl aqueous solution (60 mL). Two phases were separated, and the aqueous phase was extracted with EtOAc (60 × 3 mL). The combined organic phases were washed with brine (120 mL), and dried over anhydrous Na_2_SO_4_, filtered, and concentrated by a rotary evaporator. The residue was purified by column chromatography on silica gel with an eluent of petroleum ether/EtOAc 10:1 to afford (*R*)-4-phenyl-3-(pentadec-14-enoyl)oxazolidin-2-one ((*R*)-**4**) (7.15 g, 80% yield) as a white solid. [α${]}_{D}^{22}$ = −39.042 (c = 3.76, CHCl_3_). ^1^H NMR (500 MHz, CDCl_3_) δ 7.39–7.28 (m, 5H), 5.80 (ddt, *J* = 16.9, 10.3, 6.6 Hz, 1H), 5.42 (dd, *J* = 8.8, 3.7 Hz, 1H), 4.98 (dd, *J* = 17.1, 2.0 Hz, 1H), 4.93–4.91 (m, 1H), 4.68 (t, *J* = 8.8 Hz, 1H), 4.27 (dd, *J* = 8.9, 3.7 Hz, 1H), 2.97–2.86 (m, 2H), 2.04–2.00 (m, 2H), 1.61–1.58 (m, 2H), 1.36–1.23 (m, 10H). ^13^C NMR (126 MHz, CDCl_3_) δ 173.00, 153.87, 139.34, 139.31, 129.29, 128.80, 126.03, 114.24, 70.06, 57.68, 35.66, 33.89, 29.38, 29.15, 29.10, 29.00, 24.25. HRMS (ESI): calculated for C_20_H_28_O_3_N [M + H]^+^: 330.20637, found: 330.20667.

### 3.3. Synthesis of (R)-3-((R)-2-Methylundec-10-enoyl)-4-phenyloxazolidin-2-one ((R,R))-***5***) (New Compound)

To a 250 mL three-neck flask were added (*R*)-4-phenyl-3-(pentadec-14-enoyl)oxazolidin-2-one ((*R*)-**4**) (2.96 g, 8.98 mmol) and THF (10 mL) at room temperature. The solution was cooled to −78 °C, then NaHMDS (6.75 mL, 2.0 M in THF, 13.50 mmol) was added dropwise and stirred for 1 h. Iodomethane (6.37 g, 44.88 mmol) in THF (50 mL) was added dropwise over 2 h. After the reaction mixture was stirred for 4 h at −78 °C, it was quenched with saturated NH_4_Cl aqueous solution (40 mL). The two phases were separated, and the aqueous phase was extracted with EtOAc (50 × 3 mL). The combined organic phases were washed with brine (200 mL) and dried over anhydrous Na_2_SO_4_, filtered, and concentrated by a rotary evaporator. The residue was purified by column chromatography on silica gel with an eluent of petroleum ether/EtOAc 10:1 to afford (*R*)-3-((*R*)-2-methylundec-10-enoyl)-4-phenyloxazolidin-2-one ((*R*,*R*)-**5**) (2.69 g, 87% yield, dr > 98:2, determined by its ^13^C NMR spectra) as a white solid. m.p. 38–40 °C. [α${]}_{D}^{22}$ = −88.197 (c = 3.11, CHCl_3_). ^1^H NMR (500 MHz, CDCl_3_) δ 7.38–7.27 (m, 5H), 5.79 (dt, *J* = 16.7, 8.5 Hz, 1H), 5.43 (dd, *J* = 8.7, 3.7 Hz, 1H), 4.99 (d, *J* = 17.1 Hz, 1H), 4.92 (d, *J* = 10.2 Hz, 1H), 4.67 (t, *J* = 8.8 Hz, 1H), 4.23 (dd, *J* = 8.8, 3.7 Hz, 1H), 3.73 (h, *J* = 6.8 Hz, 1H), 2.05–2.00 (m, 2H), 1.72–1.66 (m, 1H), 1.38–1.27 (m,11H), 1.10 (d, *J* = 6.9 Hz, 3H). ^13^C NMR (126 MHz, CDCl_3_) δ 176.72, 153.46, 139.43, 139.27, 129.26, 128.70, 125.79, 114.22, 69.82, 57.81, 37.88, 33.86, 33.12, 29.67, 29.41, 29.13, 28.98, 27.35, 17.42. HRMS (ESI): calculated for C_21_H_30_O_3_N [M + H]^+^: 344.22202, found: 344.22284.

### 3.4. Synthesis of (R)-2-Methylundec-10-en-1-ol ((R)-***6***) (CAS 1156505-01-9)

To a 200 mL Schlenk flask were added (*R*)-3-((*R*)-2-methylundec-10-enoyl)-4-phenyloxazolidin-2-one ((*R*,*R*)-**5**) (1.17 g, 3.40 mmol) and THF (25 mL) at room temperature. The solution was cooled to 0 °C, then NaBH_4_ (0.52 g, 13.74 mmol) in water (2.5 mL) was added dropwise carefully. The reaction mixture was allowed to warm to room temperature, and stirred for 8 h. After being cooled to 0 °C, HCl aqueous solution (1 M) was added dropwise carefully to destroy the excess NaBH_4_. The two phases were separated, and the aqueous phase was extracted with EtOAc (20 × 3 mL). The combined organic phases were washed with brine (80 mL), and dried over anhydrous Na_2_SO_4_, filtered, and concentrated by a rotary evaporator. The residue was purified by column chromatography on silica gel with an eluent of petroleum ether/EtOAc 5:1 to afford (*R*)-2-methylpentadec-14-en-1-ol ((*R*)-**6**) (0.54 g, 86% yield, > 98:2 er, based on the signal/noise ratio of the ^19^F NMR spectra of its Mosher ester, see the details in Supplementary Materials) as a colorless oil. [α${]}_{D}^{22}$ = +8.162 (c = 1.81, CHCl_3_). Lit. [27] [α${]}_{D}^{25}$ = + 8.40 (c = 4.60, CHCl_3_). ^1^H NMR (500 MHz, CDCl_3_) δ 5.81 (ddt, *J* = 16.9, 10.2, 6.7 Hz, 1H), 4.99 (dq, *J* = 17.1, 1.8 Hz, 1H), 4.96–4.92 (m, 1H), 3.50 (dd, *J* = 10.5, 5.8 Hz, 1H), 3.41 (dd, *J* = 10.5, 6.6 Hz, 1H), 2.06–2.02 (m, 2H), 1.61–1.57 (m, 1H), 1.43–1.25 (m, 12H), 1.11 (dd, *J* = 12.1, 9.0 Hz, 1H), 0.91 (d, *J* = 6.8 Hz, 3H). ^13^C NMR (126 MHz, CDCl_3_) δ 139.35, 114.25, 68.53, 35.89, 33.93, 33.27, 30.01, 29.60, 29.25, 29.06, 27.09, 16.72. HRMS (ESI) m/z calcd. for C_12_H_23_O [M − H]^+^: 183.17434, found183.17367.

### 3.5. Synthesis of (S)-4-Phenyl-3-(undec-10-enoyl)oxazolidin-2-one((S)-***4***) (New Compound)

According to the similar procedure for oxazolidinone imide (*R*)-**4**, the acylation of undec-10-enoic acid (**2**) (6.00 g, 32.56 mmol) with (*S*)-4-phenyl-2-oxazolidinone ((*S*)-**3**) (4.71 g, 28.86 mmol) afforded (*S*)-4-phenyl-3-(undec-10-enoyl)oxazolidin-2-one ((*S*)-**4**) (9.00 g, 84% yield) as a white solid. [α${]}_{D}^{22}$ = +44.468 (c = 3.76, CHCl_3_). ^1^H NMR (500 MHz, CDCl_3_) δ 7.39–7.28 (m, 5H), 5.80 (ddt, *J* = 17.0, 10.1, 6.6 Hz, 1H), 5.42 (dd, *J* = 8.7, 3.7 Hz, 1H), 4.98 (dd, *J* = 17.1, 2.0 Hz, 1H), 4.92 (dd, *J* = 10.2, 2.2 Hz, 1H), 4.68 (t, *J* = 8.8 Hz, 1H), 4.27 (dd, *J* = 8.9, 3.7 Hz, 1H), 2.97–2.86 (m, 2H), 2.04–2.00 (m, 2H), 1.62–1.56 (m, 2H), 1.36–1.24 (m, 10H). ^13^C NMR (126 MHz, CDCl_3_) δ 172.97, 153.85, 139.33, 139.31, 129.28, 128.79, 126.02, 114.24, 70.05, 57.67, 35.65, 33.89, 29.37, 29.14, 29.09, 28.99, 24.23. HRMS (ESI): calculated for C_20_H_27_O_3_NNa [M + Na]^+^: 352.18831, found: 352.18842.

### 3.6. Synthesis of (S)-3-((S)-2-Methylundec-10-enoyl)-4-phenyloxazolidin-2-one ((S,S)-***5***) (New Compound)

According to the similar procedure for oxazolidinone imide (*R*,*R*)-**5**, the alkylation of (*S*)-4-phenyl-3-(undec-10-enoyl)oxazolidin-2-one ((*S*)-**4**) (4.00 g, 12.14 mmol) with iodomethane (8.62 g, 60.73 mmol) afforded (*S*)-3-((*S*)-2-methylundec-10-enoyl)-4-phenyloxazolidin-2-one ((*S*,*S*)-**5**) (3.13 g, 75% yield, dr > 98:2, determined by its ^13^C NMR spectra) as a white solid. m.p. 37–39 °C. [α${]}_{D}^{22}$ = +76.918 (c = 4.07, CHCl_3_). ^1^H NMR (500 MHz, CDCl_3_) δ 7.39–7.27 (m, 5H), 5.80 (ddt, *J* = 16.9, 10.1, 6.6 Hz, 1H), 5.43 (dd, *J* = 8.8, 3.7 Hz, 1H), 4.99 (dd, *J* = 8.8, 3.7 Hz, 1H), 4.92 (dd, *J* = 8.8, 3.7 Hz, 1H), 4.67 (t, *J* = 8.8 Hz, 1H), 4.24 (ddd, *J* = 8.9, 3.8, 1.0 Hz, 1H), 3.73 (h, *J* = 6.8 Hz, 1H), 2.06–2.00 (m, 2H), 1.73–1.66 (m, 1H), 1.38–1.27 (m, 11H), 1.10 (d, *J* = 6.9 Hz, 3H). ^13^C NMR (126 MHz, CDCl_3_) δ 176.77, 153.49, 139.45, 139.31, 129.30, 128.74, 125.82, 114.24, 69.85, 57.85, 37.92, 33.89, 33.16, 29.70, 29.44, 29.17, 29.01, 27.38, 17.44. HRMS (ESI): calculated for C_21_H_29_O_3_NNa [M + Na]^+^: 366.20396, found: 366.20413.

### 3.7. Synthesis of (S)-2-Methylundec-10-en-1-ol ((S)-***6***) (CAS 2091161-95-2)

According to the similar procedure for alcohol (*R*)-**6**, the reductive cleavage of (*S*)-3-((*S*)-2-methylundec-10-enoyl)-4-phenyloxazolidin-2-one ((*S*,*S*)-**5**) (2.01 g, 5.85 mmol) with NaBH_4_ (0.89 g, 23.40 mmol) afforded (*S*)-2-methylundec-10-en-1-ol ((*S*)-**6**) (0.88 g, 82% yield, >98:2 er, based on the signal/noise ratio of the ^19^F NMR spectra of its Mosher ester, see the details in Supplementary Materials) as a colorless oil. [α${]}_{D}^{22}$ = −7.156 (c = 2.18, CHCl_3_). Lit. [32] [α${]}_{D}^{25}$ = −5.1 (c = 0.85, CHCl_3,_ 82% ee). ^1^H NMR (500 MHz, CDCl_3_) δ 5.81 (ddt, *J* = 16.9, 10.2, 6.7 Hz, 1H), 4.99 (dq, *J* = 17.2, 1.8 Hz, 1H), 4.93 (ddt, *J* = 10.2, 2.4, 1.2 Hz, 1H), 3.50 (dd, *J* = 10.5, 6.2 Hz, 1H), 3.41 (dd, *J* = 10.5, 6.2 Hz, 1H), 2.06–2.02 (m, 2H), 1.63–1.59 (m, 1H), 1.39–1.24 (m, 12H), 1.13–1.09 (m, 1H), 0.91 (d, *J* = 6.7 Hz, 3H). ^13^C NMR (126 MHz, CDCl_3_) δ 139.37, 114.25, 68.55, 35.90, 33.94, 33.27, 30.01, 29.60, 29.26, 29.07, 27.10, 16.72. HRMS (ESI): calculated for C_12_H_23_ [M − OH]^+^: 167.17943, found: 167.18015.

### 3.8. Synthesis of (R)-2-Methylundec-10-en-1-yl 4-methylbenzenesulfonate ((R)-***7***) (New Compound)

To a 200 mL Schlenk flask were added (*R*)-2-methylpentadec-14-en-1-ol ((*R*)-**6**) (2.00 g, 11.40 mmol) and CH_2_Cl_2_ (20 mL) at room temperature. The solution was cooled to 0 °C, then Et_3_N (3.07 g, 30.34 mmol) and DMAP (1.32 g, 10.80 mmol) in CH_2_Cl_2_ (20 mL) were added. Subsequently, TsCl (3.93 g, 20.61 mmol) in CH_2_Cl_2_ (30 mL) was added dropwise over 30 min. After the reaction mixture was allowed to warm to room temperature and stirred for 8 h, it was quenched with saturated NH_4_Cl aqueous solution (50 mL). The two phases were separated, and the aqueous phase was extracted with CH_2_Cl_2_ (40 × 3 mL). The combined organic phases were washed with brine (150 mL) and dried over anhydrous Na_2_SO_4_, filtered, and concentrated by a rotary evaporator. The residue was purified by column chromatography on silica gel with an eluent of petroleum ether/EtOAc 10:1 to afford (*R*)-2-methylpentadec-14-en-1-yl 4-methylbenzenesulfonate ((*R*)**-7**) (3.23 g, 88% yield) as a pale yellow oil. [α${]}_{D}^{22}$ = −3.178 (c = 3.15, CHCl_3_). ^1^H NMR (500 MHz, CDCl_3_) δ 7.80–7.77 (m, 2H), 7.34 (d, *J* = 8.1 Hz, 2H), 5.81 (ddt, *J* = 16.9, 10.2, 6.6 Hz, 1H), 4.99 (dq, *J* = 17.1, 1.7 Hz, 1H), 4.93 (ddt, *J* = 10.2, 2.4, 1.3 Hz, 1H), 3.88 (dd, *J* = 9.3, 5.7 Hz, 1H), 3.80 (dd, *J* = 9.3, 6.5 Hz, 1H), 2.45 (s, 3H), 2.05–2.00 (m, 2H), 1.79–1.73 (m, 1H), 1.37–1.07 (m, 12H), 0.87 (d, *J* = 6.8 Hz, 3H). ^13^C NMR (126 MHz, CDCl_3_) δ 144.71, 139.27, 133.36, 129.90, 128.02, 114.29, 75.26, 33.89, 32.93, 32.78, 29.74, 29.47, 29.18, 29.02, 26.67, 21.75, 16.57. HRMS (ESI): calculated for C_19_H_30_O_3_NaS [M + Na]^+^: 361.18079, found: 361.18073.

### 3.9. Synthesis of (R)-10,14-Dimethylpentadec-1-ene ((R)-***9***) (New Compound)

To a 250 mL three-neck flask was added (*R*)-2-methylpentadec-14-en-1-yl 4-methylbenzenesulfonate ((*R*)-**7**) (2.95 g, 8.71 mmol) in anhydrous THF (10 mL) at room temperature. After being to 0 °C, isopentyl magnesium bromide (**8**) (26.80 mL, 1.3 M in THF, 34.84 mmol) was then added dropwise. Subsequently, a solution of Li_2_CuCl_4_ (26.10 mL, 0.1 M in THF, 2.61 mmol) was added dropwise at 0 °C. The reaction mixture was cooled to −78 °C and stirred for 1 h, then warmed to −10 °C and stirred for 2 h. After the reaction mixture was allowed to warm to 25 °C and stirred for 8 h, it was quenched with a saturated NH_4_Cl aqueous solution (80 mL) at 0 °C. The two phases were separated, and the aqueous phase was extracted with EtOAc (100 × 3 mL). The combined organic phases were washed with brine (300 mL) and dried over anhydrous Na_2_SO_4_, filtered, and concentrated by a rotary evaporator. The residue was purified by column chromatography on silica gel with an eluent of n-hexane to afford (*R*)-10,14-dimethylpentadec-1-ene ((*R*)-**9**) (1.99 g, 96% yield) as a colorless oil. [α${]}_{D}^{22}$ = +0.932 (c = 2.15, CHCl_3_). ^1^H NMR (500 MHz, CDCl_3_) δ 5.82 (ddt, *J* = 16.9, 10.2, 6.7 Hz, 1H), 4.99 (dq, *J* = 17.1, 1.8 Hz, 1H), 4.93 (ddt, *J* = 10.1, 2.3, 1.3 Hz, 1H), 2.06–2.02 (m, 2H), 1.56–1.48 (m, 1H), 1.39–1.22 (m, 19H), 0.86 (d, *J* = 6.6 Hz, 6H), 0.83 (d, *J* = 6.6 Hz, 3H). ^13^C NMR (126 MHz, CDCl3) δ 139.44, 114.22, 39.53, 37.48, 37.25, 33.98, 32.92, 30.11, 29.70, 29.32, 29.12, 28.14, 27.22, 27.20, 24.95, 22.87, 22.78, 19.87. HRMS (ESI): calculated for C_17_H_36_O [M + H_2_O]^+^: 256.27607, found: 256.27584.

### 3.10. Synthesis of (R)-10,14-Dimethylpentadecan-1-ol ((R)-***10***) (CAS 164260-11-1)

To a 50 mL Schlenk tube were added (*R*)-10,14-dimethylpentadec-1-ene ((*R*)-**9**) (1.30 g, 5.45 mmol) and anhydrous THF (10 mL) at room temperature. The resulting solution was cooled to 0 °C, then borane (1.82 mL, 1.0 M in THF, 1.82 mmol) was added dropwise over 20 min. The reaction mixture was warmed to room temperature and stirred for 1 h, and the solution of tris((*S*)-10,14-dimethylpentadecyl)borane was obtained. Then the solution was cooled to 0 °C, and sodium hydroxide (0.61 mL, 3 N, 1.82 mmol) was added. The resulting solution was cooled to −20 °C, and hydrogen peroxide (0.65 mL, 30%, 6.32 mmol) was added dropwise. After the reaction was warmed to 35 °C and stirred for 1 h, it was quenched with water (10 mL). The two phases were separated, and the aqueous phase was extracted with EtOAc (15 × 3 mL). The combined organic phases were washed with brine (20 mL) and dried over anhydrous Na_2_SO_4_, filtered, and concentrated by a rotary evaporator. The residue was purified by column chromatography on silica gel with an eluent of petroleum ether/EtOAc 5:1 to afford (*R*)-10,14-dimethylpentadecan-1-ol ((*R*)-**10**) (0.77 g, 55% yield) as a pale yellow oil. [α${]}_{D}^{22}$ = + 0.556 (c = 1.44, CHCl_3_). ^1^H NMR (500 MHz, CDCl_3_) δ 3.64 (t, *J* = 6.6 Hz, 2H), 1.59–1.49 (m, 3H), 1.35–1.32 (m, 1H), 1.29–1.20 (m, 17H), 1.15–1.11 (m, 2H), 1.09–1.03 (m, 2H), 0.87 (d, *J* = 6.7 Hz, 6H), 0.84 (d, *J* = 6.6 Hz, 3H). ^13^C NMR (126 MHz, CDCl_3_) δ 63.25, 39.52, 37.47, 37.25, 32.97, 32.91, 30.15, 29.79, 29.78, 29.59, 28.13, 27.23, 25.89, 24.95, 22.87, 22.77, 19.86. HRMS (ESI): calculated for C_17_H_37_O [M + H]^+^: 257.28389, found: 257.28122.

### 3.11. Synthesis of (S)-2-Methylundec-10-en-1-yl 4-methylbenzenesulfonate ((S)-***7***) (New Compound)

According to the similar procedure for tosylate (*R*)-**7**, the tosylation of (*S*)-2-methylpentadec-14-en-1-ol ((*S*)-**6**) (1.50 g, 8.14 mmol) with TsCl (2.81 g, 14.72 mmol) afforded (*S*)-2-methylundec-10-en-1-yl 4-methylbenzenesulfonate ((*S*)-**7**) (2.42 g, 88% yield) as a pale yellow oil. [α${]}_{D}^{22}$ = +3.734 (c = 1.61, CHCl_3_). ^1^H NMR (500 MHz, CDCl_3_) δ 7.79 (d, *J* = 8.3 Hz, 2H), 7.34 (d, *J* = 8.0 Hz, 2H), 5.81 (ddt, *J* = 16.9, 10.2, 6.7 Hz, 1H), 4.99 (dq, *J* = 17.1, 1.7 Hz, 1H), 4.93 (ddt, *J* = 10.2, 2.3, 1.3 Hz, 1H), 3.88 (dd, *J* = 9.3, 5.7 Hz, 1H), 3.80 (dd, *J* = 9.4, 6.5 Hz, 1H), 2.45 (s, 3H), 2.05–2.00 (m, 2H), 1.78–1.74 (m, 1H), 1.37–1.10 (m, 12H), 0.87 (d, *J* = 6.8 Hz, 3H). ^13^C NMR (126 MHz, CDCl_3_) δ 144.71, 139.28, 133.36, 129.90, 128.02, 114.29, 75.26, 33.90, 32.94, 32.78, 29.74, 29.48, 29.18, 29.02, 26.67, 21.76, 16.57. HRMS (ESI): calculated for C_19_H_32_O_4_S [M + H_2_O]^+^: 356.20158, found: 356.20156.

### 3.12. Synthesis of (S)-10,14-Dimethylpentadec-1-ene ((S)-***9***) (New Compound)

According to the similar procedure for alkene (*R*)-**9**, the coupling of (*S*)-2-methylpentadec-14-en-1-yl 4-methylbenzenesulfonate ((*S*)**-7**) (2.02 g, 5.97 mmol) with isopentyl magnesium bromide (**8**) (18.38 mL, 1.3 M in THF, 23.88 mmol) catalyzed by Li_2_CuCl_4_ (18.0 mL, 0.1 M in THF, 1.79 mmol) afforded (*S*)-10,14-dimethylpentadec-1-ene ((*S*)-**9**) (1.28 g, 90% yield) as a colorless oil. [α${]}_{D}^{22}$ = −0.631 (c = 1.27, CHCl_3_). ^1^H NMR (500 MHz, CDCl_3_) δ 5.81 (ddt, *J* = 16.9, 10.2, 6.7 Hz, 1H), 4.99 (dq, *J* = 17.1, 1.7 Hz, 1H), 4.94–4.91 (m, 1H), 2.06–2.02 (m, 2H), 1.55–1.50 (m, 1H), 1.39–1.15 (m, 19H), 0.87 (d, *J* = 6.9 Hz, 6H), 0.83 (d, *J* = 6.6 Hz, 3H). ^13^C NMR (126 MHz, CDCl_3_) δ 139.41, 114.24, 39.55, 37.50, 37.27, 34.00, 32.94, 30.13, 29.72, 29.34, 29.14, 28.15, 27.24, 24.98, 22.88, 22.79, 19.88. HRMS (ESI): calculated for C_17_H_36_O [M + H_2_O]^+^: 256.27607, found: 256.27575.

### 3.13. Synthesis of (S)-10,14-Dimethylpentadecan-1-ol ((S)-***10***) (CAS 164260-12-2)

According to the similar procedure for alcohol (*R*)-**10**, the hydroboration-oxidation of (*S*)-10,14-dimethylpentadec-1-ene ((*S*)-**9**) (1.26 g, 5.28 mmol) with borane (1.76 mL, 1.0 M in THF, 1.76 mmol) and hydrogen peroxide (0.63 mL, 30%, 6.12 mmol) afforded (*S*)-10,14-dimethylpentadecan-1-ol ((*S*)-**10**) (0.73 g, 54% yield) as a colorless oil. [α${]}_{D}^{22}$ = −0.214 (c = 3.74, CHCl_3_). ^1^H NMR (500 MHz, CDCl_3_) δ 3.63 (t, *J* = 6.7 Hz, 2H), 1.59–1.54 (m, 2H), 1.52–1.48 (m, 1H), 1.45 (br s, 1H), 1.36–1.20 (m, 17H), 1.15–1.11 (m, 2H), 1.09–1.03 (m, 2H), 0.87 (d, *J* = 6.6 Hz, 6H), 0.84 (d, *J* = 6.6 Hz, 3H). ^13^C NMR (126 MHz, CDCl_3_) δ 63.20, 39.51, 37.46, 37.25, 32.96, 32.91, 30.15, 29.79, 29.77, 29.59, 28.12, 27.22, 25.89, 24.94, 22.85, 22.76, 19.85. HRMS (ESI): calculated for C_17_H_35_ [M − OH]^+^: 239.27333, found: 239.27253.

### 3.14. Synthesis of (R)-10,14-Dimethylpentadecyl Isobutyrate ((R)-***1***) (164260-03-1)

To a 20 mL Schlenk tube were added (*R*)-10,14-dimethylpentadecan-1-ol ((*R*)-**10**) (0.40 g, 1.56 mmol) and DMAP (0.19 g, 1.56 mmol) in CH_2_Cl_2_ (5 mL) at room temperature. Isobutyric anhydride (**11**) (0.37 g, 2.34 mmol) was then added dropwise. After the reaction solution was stirred for 9 h at the same temperature, it was diluted with CH_2_Cl_2_ (5 mL). The organic solution was washed with HCl aqueous solution (1 N, 20 mL), NaOH aqueous solution (1 N, 20 mL), NaHCO_3_ solution (20 mL) and brine (20 mL) sequentially. Finaly, the organic solution was dried over anhydrous Na_2_SO_4_ and concentrated by a rotary evaporator to afford (*R*)-10,14-dimethylpentadecyl isobutyrate ((*R*)-**1**) (0.47 g, 93% yield) as a pale yellow oil. [α${]}_{D}^{22}$ = +1.148 (c = 2.79, CHCl_3_). Lit. [19] [α${]}_{D}^{23}$ = + 0.581 (c = 2.3, CHCl_3_). ^1^H NMR (500 MHz, CDCl_3_) δ 4.06 (t, *J* = 6.7 Hz, 2H), 2.53 (h, *J* = 7.0 Hz, 1H), 1.65–1.59 (m, 2H), 1.56–1.48 (m, 1H), 1.36–1.22 (m, 18H), 1.16 (d, *J* = 7.0 Hz, 6H), 1.14–1.11 (m, 1H), 1.10–1.03 (m, 2H), 0.87 (d, *J* = 6.6 Hz, 6H), 0.84 (d, *J* = 6.6 Hz, 3H). ^13^C NMR (126 MHz, CDCl_3_) δ 177.42, 64.56, 39.52, 37.47, 37.25, 34.20, 32.91, 30.13, 29.75, 29.68, 29.40, 28.80, 28.13, 27.22, 26.05, 24.95, 22.86, 22.77, 19.86, 19.17. HRMS (ESI): calculated for C_21_H_42_O_2_KNa [M + K + Na]^+^: 388.27141, found: 388.27005.

### 3.15. Synthesis of (S)-10,14-Dimethylpentadecyl Isobutyrate ((S)-***1***) (CAS164260-04-2)

According to the similar procedure for the sex pheromone (*R*)-**1**, the esterification of (*S*)-10,14-dimethylpentadecan-1-ol ((*S*)-**10**) (0.30 g, 1.17 mmol) with isobutyric anhydride (**11**) (0.28 g, 1.77 mmol) afforded (*S*)-10,14-dimethylpentadecyl isobutyrate ((*S*)-**1**) (0.34 g, 90% yield) as a pale yellow oil. [α${]}_{D}^{22}$ = −0.563 (c = 1.42, CHCl_3_). Lit. [18] [α${]}_{D}^{25}$ = −0.28 (c = 2.40, CHCl_3_). ^1^H NMR (500 MHz, CDCl_3_) δ 4.06 (t, *J* = 6.7 Hz, 2H), 2.53 (h, *J* = 7.0 Hz, 1H), 1.65–1.59 (m, 2H), 1.56–1.48 (m, 1H), 1.37–1.21 (m, 18H), 1.16 (d, *J* = 7.0 Hz, 6H), 1.14–1.11 (m, 1H), 1.09–1.05 (m, 2H), 0.86 (d, *J* = 6.6 Hz, 6H), 0.84 (d, *J* = 6.6 Hz, 3H). ^13^C NMR (126 MHz, CDCl_3_) δ 177.38, 64.54, 39.51, 37.46, 37.24, 34.19, 32.91, 30.12, 29.75, 29.67, 29.40, 28.80, 28.12, 27.21, 26.05, 24.94, 22.85, 22.76, 19.85, 19.15. HRMS (ESI): calculated for C_21_H_41_O_2_ [M − H]^+^: 325.31011, found: 325.30765.

## 4. Conclusions

In summary, we have developed a new and concise asymmetric synthesis of the sex pheromone of *E. pseudoconspersa*, (*R*)-10,14-dimethylpentadecyl isobutyrate ((*R*)-**1**), and its enantiomer. The key reactions of our strategy involved Evans’s chiral auxiliaries, the coupling of chiral tosylate with Grignard reagent, and the esterification of chiral alcohol. Moreover, the synthetic pheromone would be useful to control the tea tussock moth.

## Data Availability

The data presented in this article are available in the Supplementary Materials.

## Supplementary Materials

The following supporting information can be downloaded at: https://www.mdpi.com/article/10.3390/molecules30122494/s1. Research on the Optical Purity of the Chiral Alcohols (R)- and (S)-6, ^1^H and ^13^C NMR Spectra of the Products. Refs. [33,34] are cited in the Supplementary Materials.
