# Supplementary material for: A Concise Asymmetric Synthesis of Sex Pheromone of Euproctis pseudoconspersa (Strand) and Its Enantiomer
Source: Molecules. 2025 Jun 6;30(12):2494. doi: 10.3390/molecules30122494 (PMC12195855; doi:10.3390/molecules30122494)
Supplement: Supplementary file 1 [file molecules-30-02494-s001.zip › molecules-3659669-supplementary.pdf]

SUPPORTING INFORMATION

A Concise Asymmetric Synthesis of Sex Pheromone of *Euproctis pseudoconspersa* (Strand) and Its Enantiomer

Biyu An, Shengli Liu, Jianan Wang, Dan Liu, Qinghua Bian * and Jiangchun Zhong *

Department of Applied Chemistry, China Agricultural University, Beijing 100193, China;
aby19990129@163.com (B.A.); xxjnwang@163.com (J.W.)
* Correspondence: bianqinghua@cau.edu.cn (Q.B.); zhong@cau.edu.cn (J.Z.)

.

Table of Contents

1. General Information………………………………………………………………….…….S1

2. Research on the Optical Purity of the Chiral Alcohols (*R*)- and (*S*)-**6**………… …………S2

3. ^{1}H and ^{13}C NMR Spectra of the Products……………………………………………………S6

4. References…………………………………………………………………………………….S27

1. General Information

Unless otherwise stated, all reactions were conducted in oven-dried glassware with a Schlenk line under an argon atmosphere, and all reaction mixtures were magnetically stirred. Tetrahydrofuran, triethylamine, and diethyl ether were distilled from CaH_2 before use, and all starting materials and reagents were purchased from commercial sources. Optical rotations were determined by a Rudolph AUTOPOL-IV polarimeter, with a 0.25 dm cell length. ^1H and ^{13}C NMR spectra were recorded on a Bruker AscendTM 500 MHz spectrometer using deuterated solvent. Chemical shifts were reported in parts per million (ppm) with an internal standard of tetramethylsilane (0.00 ppm) for ^1H NMR spectra and the residual protium $CDCl_3$ (77.16 ppm) for ^{13}C NMR spectra. High-resolution mass spectrum (HRMS) analyses were performed on the Waters LCT Premier™ ESI mass spectrometer.

2. Research on the Optical Purity of the Chiral Alcohols (*R*)- and (*S*)-6

Scheme S1. Synthesis of racemate alcohol *rac*-**6**.

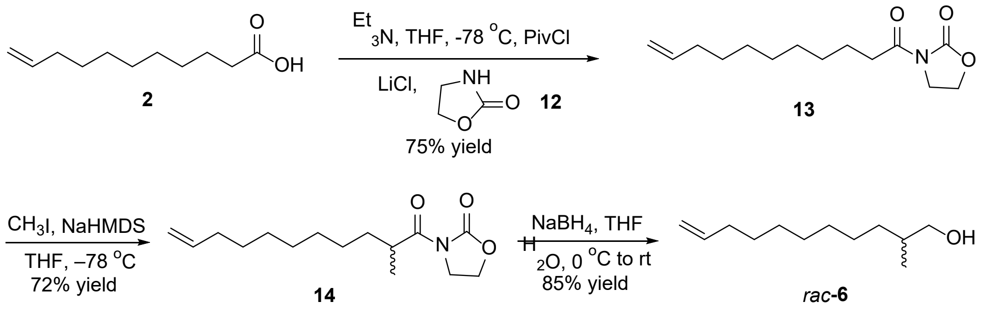

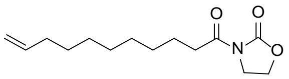

3-(undec-10-enoyl)oxazolidin-2-one (13) (CAS 2243012-31-7) [1,2]

To a 500 mL three-neck flask, undec-10-enoic acid (**2**) (5.00 g, 27.13 mmol), Et_3N (5.77 g, 56.97 mmol), and THF (150 mL) were added at room temperature. The solution was cooled to −78 °C, then pivaloyl chloride (3.93 g, 32.55 mmol) was added and the solution was stirred for 0.5 h. The reaction solution was allowed to warm to room temperature and stirred for 1 h. 2-Oxazolidinone (**12**) (2.60 g, 29.86 mmol) in THF (40 mL) and LiCl (3.45 g, 81.39 mmol) were then added. The reaction mixture was cooled to −78°C again and stirred for 1 h. After the reaction mixture was allowed to warm to room temperature and stirred for an additional 10 h, it was quenched with saturated NH_4Cl aqueous solution (60 mL). Two phases were separated, and the aqueous phase was extracted with EtOAc (60 × 3 mL). The combined organic phases were washed with brine (120 mL), then dried over anhydrous Na_2SO_4, filtered, and concentrated by a rotary evaporator. The residue was purified by column chromatography on silica gel with an eluent of petroleum ether/EtOAc 10:1 to yield 3-(undec-10-enoyl)oxazolidin-2-one (**13**) (5.15 g, 75 % yield) as a white solid. ^1H NMR (500 MHz, $CDCl_3$) δ 5.74 (dd, *J* = 17.1, 8.7 Hz, 1H), 4.93 – 4.84 (m, 2H), 4.33 (t, *J* = 8.2 Hz, 2H), 3.95 (t, *J* = 8.1 Hz, 2H), 2.83 (d, *J* = 7.4 Hz, 2H), 1.99 – 1.94 (m, 2H), 1.59 – 1.55 (m, 2H), 1.31 – 1.18 (m, 10H). ^{13}C NMR (126 MHz, $CDCl_3$) δ 173.69, 153.65, 139.30, 114.23, 62.10, 42.63, 35.20, 33.88, 29.39, 29.20, 29.16, 29.00, 24.35. HRMS (ESI): calculated for $C_{14}H_{23}O_3NNa$ $[M+Na]^+$: 276.15701, found: 276.15689.

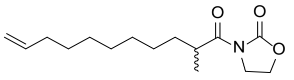

3-(2-methylundec-10-enoyl)oxazolidin-2-one (14) (New compound) [3,4]

To a 250 mL three-neck flask, 3-(undec-10-enoyl)oxazolidin-2-one (**13**) (2.50 g, 9.87 mmol), and THF (10 mL) were added at room temperature. The solution was cooled to −78 °C, then NaHMDS (7.39 mL, 2.0 M in THF, 14.79 mmol) was added dropwise and stirred for 1 h. Iodomethane (7.00 g, 49.32 mmol) in THF (50 mL) was added dropwise over 2 h. After the reaction mixture had been stirred for 4 h at −78 °C, it was quenched with saturated NH_4Cl aqueous solution (40 mL). Two phases were separated, and the aqueous phase was extracted with EtOAc (50 × 3 mL). The combined organic phases were washed with brine (200 mL), then dried over anhydrous Na_2SO_4, filtered, and concentrated by a rotary evaporator. The residue was purified by column chromatography on silica gel with an eluent of petroleum ether/EtOAc 10:1 to yield 3-(2-methylundec-10-enoyl)oxazolidin-2-one (**14**) (1.90 g, 72% yield) as a white solid. ^{1}H NMR (500 MHz, $CDCl_3$) δ 5.80 (td, *J* = 16.8, 6.8 Hz, 1H), 4.99 (d, *J* = 17.1 Hz, 1H), 4.92 (d, *J* = 10.2 Hz, 1H), 4.40 (t, *J* = 8.1 Hz, 2H), 4.02 (t, *J* = 8.2 Hz, 2H), 3.72 (h, *J* = 6.8, 6.3 Hz, 1H), 2.03 (q, *J* = 7.2 Hz, 2H), 1.74 – 1.69 (m, 1H), 1.40 – 1.28 (m, 11H), 1.16 (d, *J* = 6.9 Hz, 3H). ^{13}C NMR (126 MHz, $CDCl_3$) δ 177.59, 153.27, 139.25, 114.19, 61.90, 42.88, 37.40, 33.84, 33.67, 29.62, 29.39, 29.11, 28.96, 27.20, 17.06. HRMS (ESI): calculated for $C_{15}H_{26}O_3N$ $[M+H]^+$: 268.19140, found: 268.19072.

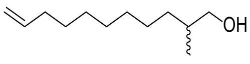

2-methylundec-10-en-1-ol (*rac*-6) (CAS 58196-28-4) [5]

To a 200 mL Schlenk flask, added 3-(2-methylundec-10-enoyl)oxazolidin-2-one (**14**) (1.17 g, 4.38 mmol) and THF (25 mL) were added at room temperature. The solution was cooled to 0 °C, then $NaBH_4$ (0.66 g, 17.45 mmol) in water (2.5 mL) was carefully added dropwise. The reaction mixture was allowed to warm to room temperature and stirred for 8 h. After being cooled to 0 °C, HCl aqueous solution (1 M) was carefully added dropwise to destroy the excess $NaBH_4$. Two phases were separated, and the aqueous phase was extracted with EtOAc (20 × 3 mL). The combined organic phases were washed with brine (80 mL), then dried over anhydrous Na_2SO_4, filtered, and concentrated by a rotary evaporator. The residue was purified by column chromatography on silica gel with an eluent of petroleum ether/EtOAc 5:1 to yield 2-methylundec-10-en-1-ol (*rac*-**6**) (0.68 g, 85% yield) as a colorless oil. ^{1}H NMR (500 MHz, $CDCl_3$) δ 5.81 (ddt, *J* = 16.9, 10.2, 6.6 Hz, 1H), 4.99 (dd, *J* = 17.1, 2.0 Hz, 1H), 4.93 (dd, *J* = 10.3, 2.1 Hz, 1H), 3.50 (dd, *J* = 10.5, 6.1 Hz, 1H), 3.40 (dd, *J* = 10.5, 6.1 Hz, 1H). ^{13}C NMR (126 MHz, $CDCl_3$) δ 139.33, 114.23, 68.49, 35.88, 33.92, 33.26, 30.00, 29.59, 29.25, 29.05, 27.08, 16.71. HRMS (ESI): calculated for $C_{12}H_{25}ONa$ $[M+Na+H]^+$: 208.17976, found: 208.17761.

Scheme S2. Synthesis of Mosher esters **16**-**18**.

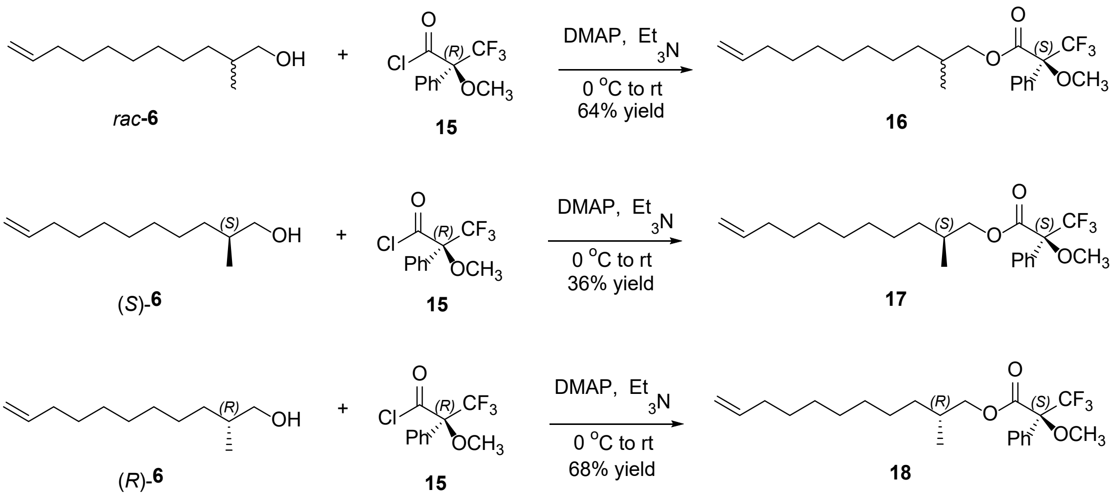

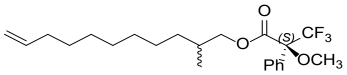 **2-methylundec-10-en-1-yl (2*S*)-3,3,3-trifluoro-2-methoxy-2-phenylpropanoate (16) (CAS 2892417-77-3) [6,7]**

To a 10 mL Schlenk tube, DMAP (0.020 g, 0.16 mmol) in CH_2Cl_2 (3 mL) and 2-methylundec-10-en-1-ol (*rac*-**6**) (0.030 g, 0.16 mmol) in CH_2Cl_2 (2 mL) were added at room temperature. The resulting solution was cooled to 0 °C, then Et_3N (0.082 g, 0.81 mmol) and (*R*)-(-)-MTPACl (**15**) (0.041 g, 0.16 mmol) in CH_2Cl_2 (1 mL) were added dropwise. After the reaction mixture had been allowed to warm to room temperature and stirred for 8 h, it was quenched with saturated NH_4Cl aqueous solution (5 mL). Two phases were separated, and the aqueous phase was extracted with EtOAc (10 × 3 mL). The combined organic phases were washed with brine (15 mL), then dried over anhydrous Na_2SO_4, filtered, and concentrated by a rotary evaporator. The residue was purified by column chromatography on silica gel with an eluent of petroleum ether/EtOAc 10:1 to afford 2-methylundec-10-en-1-yl (2*S*)-3,3,3-trifluoro-2-methoxy-2-phenylpropanoate (**16**) (0.042 g, 64% yield) as a colorless oil. ^{1}H NMR (500 MHz, $CDCl_3$) δ 7.53 – 7.51 (m, 2H), 7.41 – 7.38 (m, 3H), 5.85 – 5.77 (m, 1H), 4.99 (dd, *J* = 17.1, 1.9 Hz, 1H), 4.93 (dd, *J* = 10.1, 2.0 Hz, 1H), 4.23 (dd, *J* = 10.7, 5.6 Hz, 0.5H), 4.15 (dd, *J* = 6.1, 2.4 Hz, 1H), 4.07 (dd, *J* = 10.6, 6.7 Hz, 0.5H), 3.55 (s, 3H), 2.06 – 2.01 (m, 2H), 1.84 (q, *J* = 6.4 Hz, 1H), 1.38 – 1.23 (m, 12H), 0.91 (d, *J* = 6.8 Hz, 1.5H), 0.90 (d, *J* = 6.8 Hz, 1.5H). ^{13}C NMR (126 MHz, $CDCl_3$) δ 166.83, 139.32, 132.56, 129.71, 128.53, 127.51,123.51 (q, *J* = 289.09 Hz), 114.30, 84.79 (q, *J* = 28.20 Hz), 71.40, 71.36, 55.55, 33.92, 33.21, 33.17, 32.57, 32.53, 29.81, 29.51, 29.21, 29.04, 26.80, 16.93, 16.90. ^{19}F NMR (471 MHz, $CDCl_3$) δ -71.55, – 71.57. HRMS (ESI): calculated for $C_{22}H_{31}O_3F_3$ $[M]^+$: 400.22198, found: 400.21981. The spectral data correspond to a 1:1 mixture of diastereomeric esters, and the region between 4.00 and 4.30 ppm of the ^{1}H NMR spectra was used to determine the enantiomeric excess.

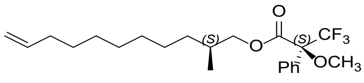 **(*S*)-2-methylundec-10-en-1-yl (*S*)-3,3,3-trifluoro-2-methoxy-2-phenylpropanoate (17) (CAS 2892417-78-4)**

According to the similar procedure for Mosher ester **16**, the esterification of (*S*)-2-methylundec-10-en-1-ol ((*S*)-**6**) (0.03 g, 0.16 mmol) with (*R*)-(-)-MTPACl (**15**) (0.041 g, 0.16 mmol) yielded (*S*)-2-methylpentadec-14-en-1-yl (*S*)-3,3,3-trifluoro-2-methoxy-2-phenylpropanoate (**17**) (0.023 g, 36% yield) as a colorless oil. $[\alpha]_D^{22}$ = –32.526 (c = 1.27, $CHCl_3$). ^{1}H NMR (500 MHz, $CDCl_3$) δ 7.53 –7.51(m, 2H), 7.41–7.38 (m, 3H), 5.83 – 5.78 (m, 1H), 4.99 (dd,

J = 17.1, 1.9 Hz, 1H), 4.93 (dd, J = 10.2, 1.1 Hz, 1H), 4.23 (dd, J = 10.7, 5.6 Hz, 1H), 4.07 (dd, J = 10.6, 6.7 Hz, 1H), 3.55 (s, 3H), 2.06 – 2.01 (m, 2H), 1.83 (q, J = 5.3 Hz, 1H), 1.38 – 1.24 (m, 12H), 0.91 (d, J = 6.7 Hz, 3H). ^{13}C NMR (126 MHz, $CDCl_3$) δ 166.82, 139.32, 129.71, 128.52, 127.51,123.51 (q, J = 289.08 Hz), 114.30, 84.78 (q, J = 27.63 Hz), 71.40, 55.55, 33.93, 33.21, 32.53, 29.82, 29.51, 29.21, 29.04, 26.80, 16.90. ^{19}F NMR (471 MHz, $CDCl_3$) δ – 71.55. HRMS (ESI) m/z calculated for $C_{22}H_{32}O_3F_3$ [M+H]$^+$: 401.22981, found: 401.23341.

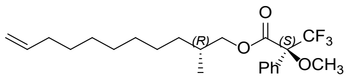

(*R*)-2-methylundec-10-en-1-yl (*S*)-3,3,3-trifluoro-2-methoxy-2-phenylpropanoate (18) (New compound)

According to the similar procedure for Mosher ester **16**, the esterification of (*R*)-2-methylundec-10-en-1-ol ((*R*)-**6**) (0.024 g, 0.13 mmol) with (*R*)-(-)-MTPACl (**15**) (0.033 g, 0.13 mmol) yielded (*R*)-2-methylpentadec-14-en-1-yl (*S*)-3,3,3-trifluoro-2-methoxy-2-phenylpropanoate (**18**) (0.036 g, 68% yield) as a colorless oil. $[\alpha]_D^{22}$ = –31.538 (c = 1.56, $CHCl_3$). ^{1}H NMR (500 MHz, $CDCl_3$) δ 7.53 – 7.51 (m, 2H), 7.41 – 7.39 (m, 3H), 5.80 (ddt, J = 16.9, 10.1, 6.6 Hz, 1H), 4.99 (dd, J = 17.2, 1.8 Hz, 1H), 4.93 (dd, 1H), 4.15 (dd, J = 6.1, 2.4 Hz, 2H), 3.55 (s, 3H), 2.06 – 2.01 (m, 2H), 1.84 (q, J = 6.1 Hz, 1H), 1.37 – 1.24 (m, 12H), 0.91 (d, J = 6.7 Hz, 3H). ^{13}C NMR (126 MHz, $CDCl_3$) δ 166.83, 139.33, 132.55, 129.72, 128.53, 127.50, 123.51 (q, J = 289.22 Hz) 114.30, 84.78 (q, J = 27.68 Hz), 71.36, 55.55, 33.93, 33.17, 32.56, 29.81, 29.50, 29.21, 29.04, 26.81, 16.92. ^{19}F NMR (471 MHz, $CDCl_3$) δ – 71.57. HRMS (ESI) m/z calculated for $C_{22}H_{30}O_3F_3$ [M-H]$^+$: 399.21470, found: 399.21416.

3. ^{1}H and ^{13}C NMR Spectra of the Products

Figure S1. ^{1}H NMR spectrum of (*R*)-4-phenyl-3-(undec-10-enoyl)oxazolidin-2-one (*R*)-**4** (500 MHz, $CDCl_3$).

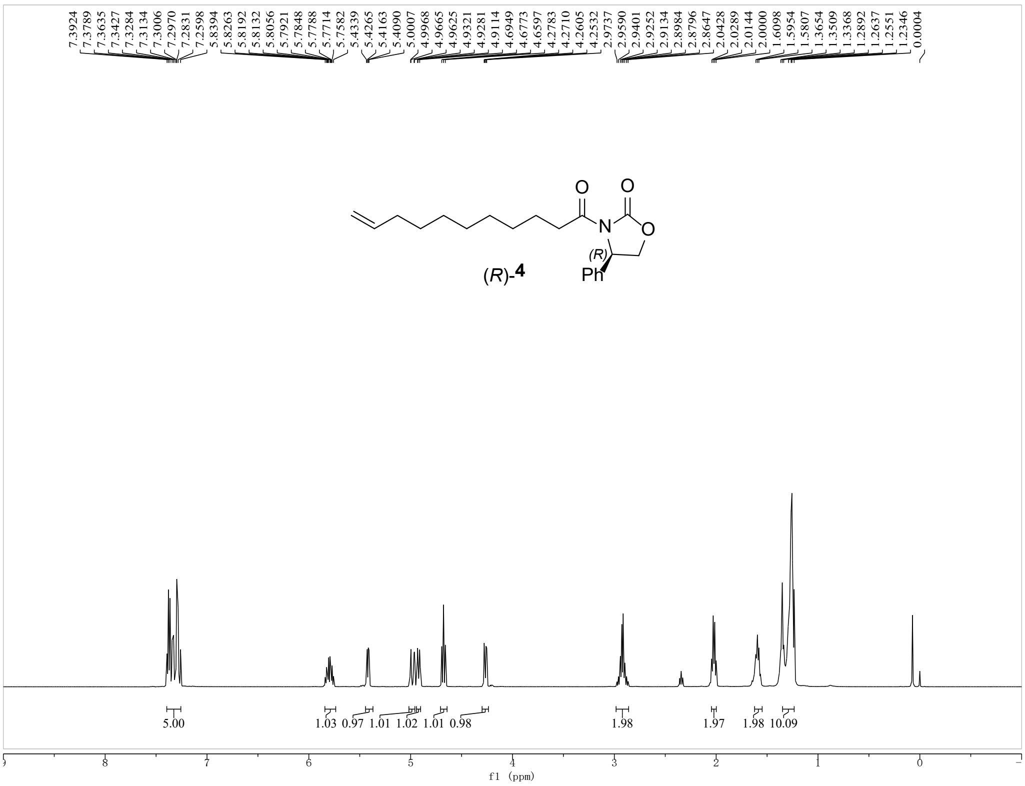

Figure S2. ^{13}C NMR spectrum of (*R*)-4-phenyl-3-(undec-10-enoyl)oxazolidin-2-one (*R*)-**4** (126 MHz, $CDCl_3$).

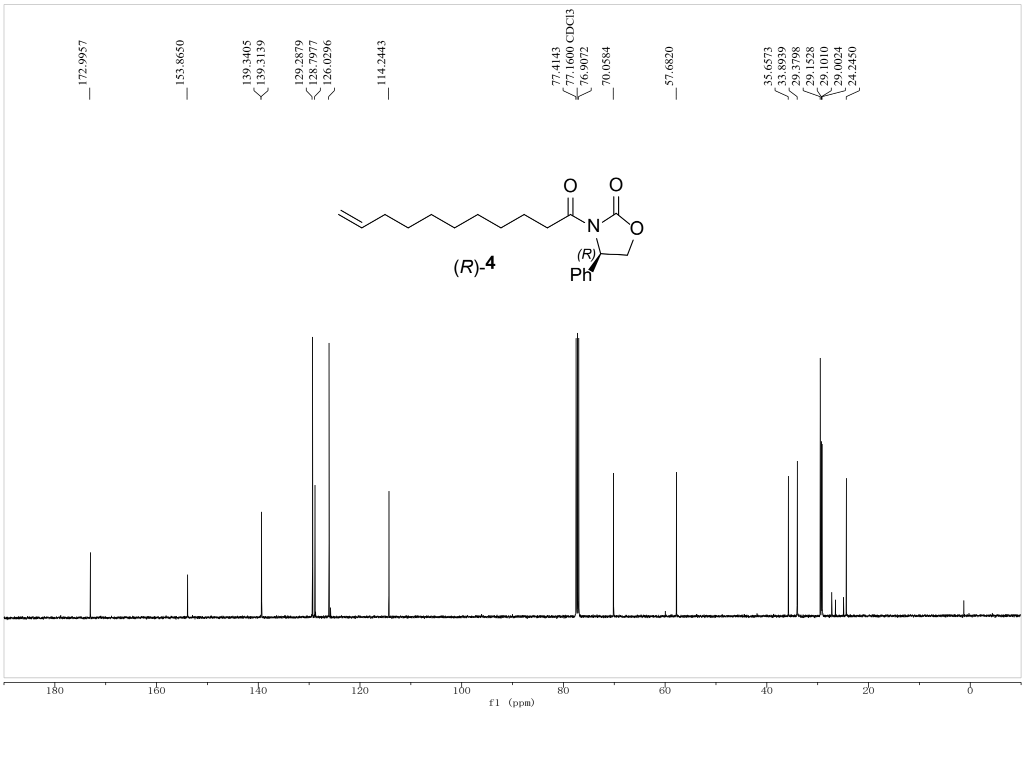

Figure S3. ^{1}H NMR spectrum of (*R*)-3-((R)-2-methylundec-10-enoyl)-4-phenyloxazolidin-2-one ((*R*,*R*)-**5**) (500 M Hz, CDCl$_3$).

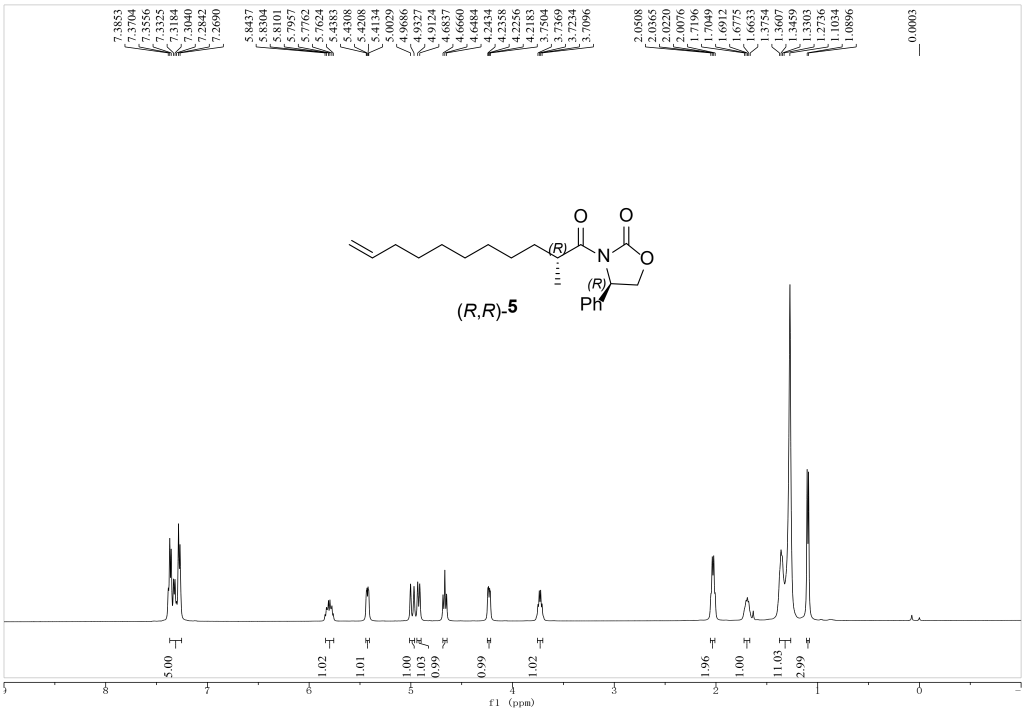

Figure S4. ^{13}C NMR (*R*)-3-((*R*)-2-methylundec-10-enoyl)-4-phenyloxazolidin-2-one ((*R*,*R*)-**5**) (126 MHz, CDCl$_3$).

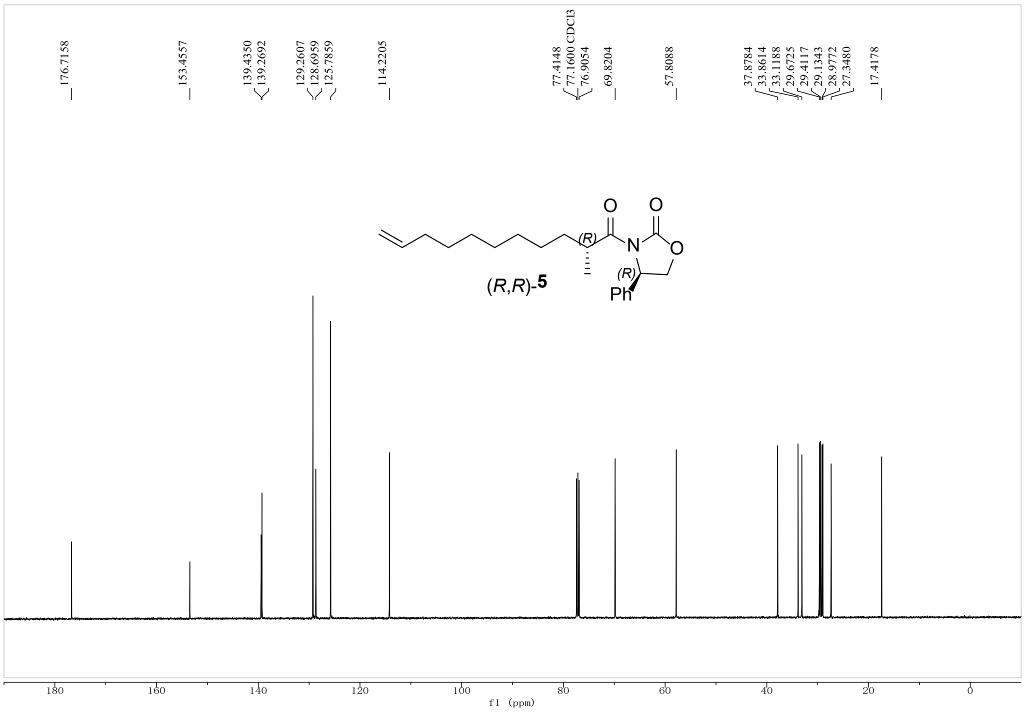

Figure S5. ^{1}H NMR spectrum of (*R*)-2-methylundec-10-en-1-ol (*R*)-**6** (500 MHz, $CDCl_3$).

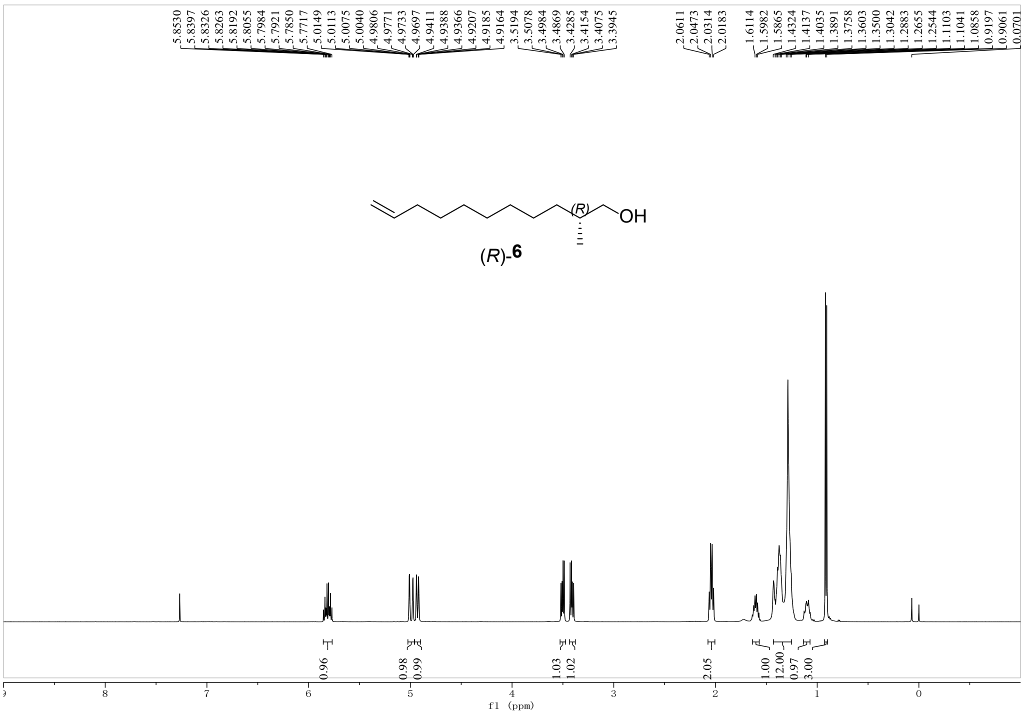

Figure S6. ^{13}C NMR spectrum of (*R*)-2-methylundec-10-en-1-ol (*R*)-**6** (126 MHz, $CDCl_3$).

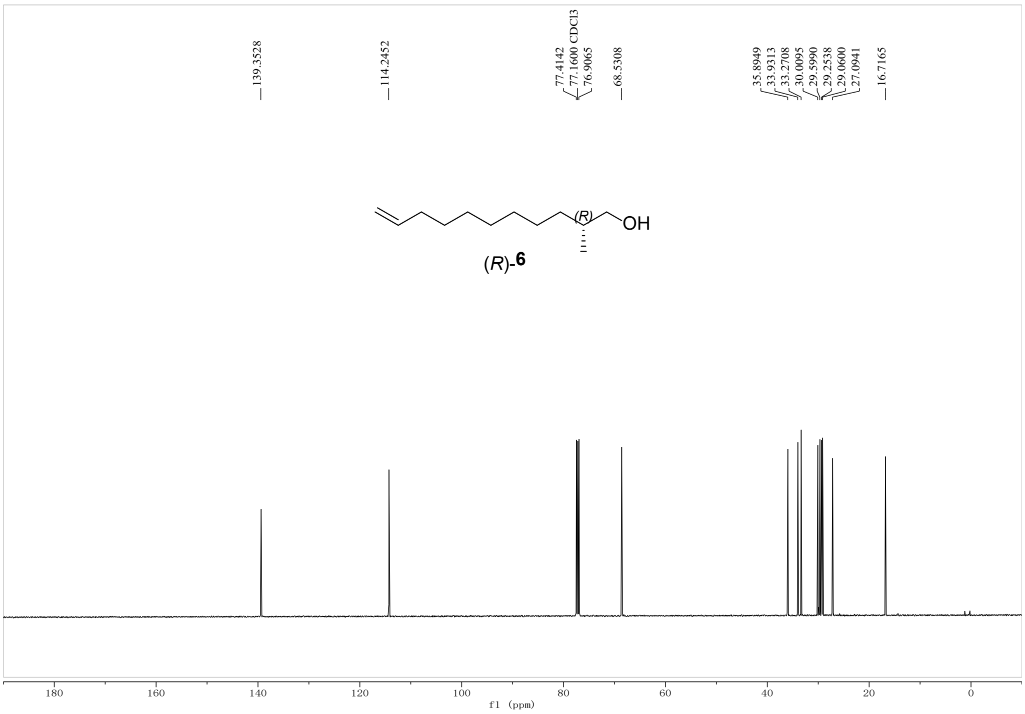

Figure S7. ^{1}H NMR spectrum of (*S*)-4-phenyl-3-(undec-10-enoyl)oxazolidin-2-one((*S*)-**4**) (500 MHz, $CDCl_3$).

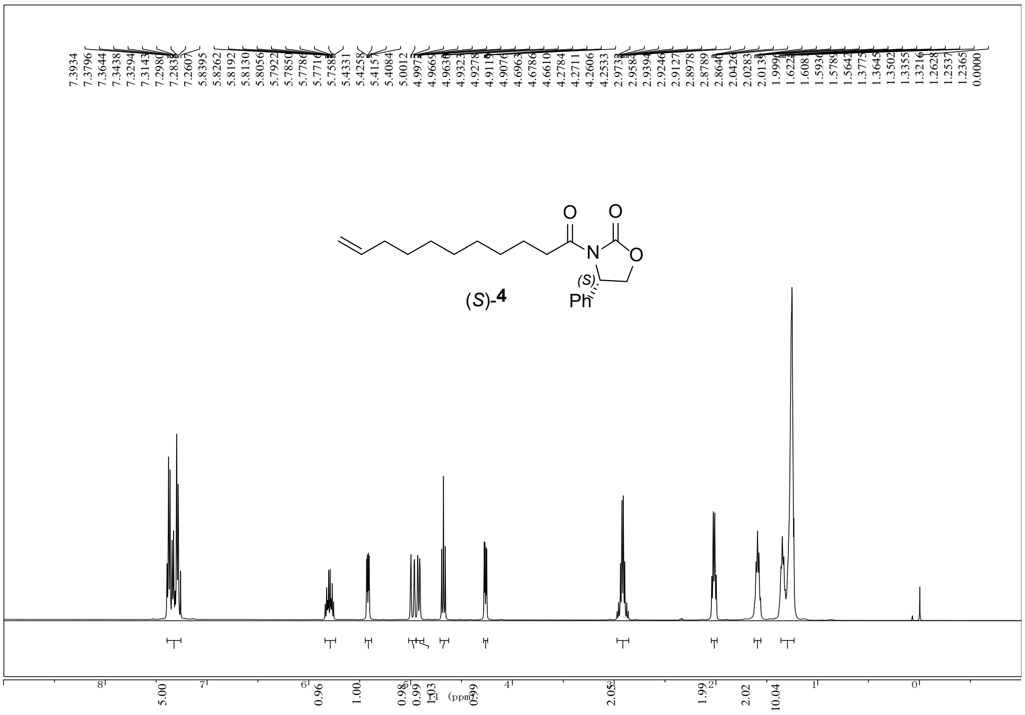

Figure S8. ^{13}C NMR spectrum of (*S*)-4-phenyl-3-(undec-10-enoyl)oxazolidin-2-one((*S*)-**4**) (126 MHz, $CDCl_3$).

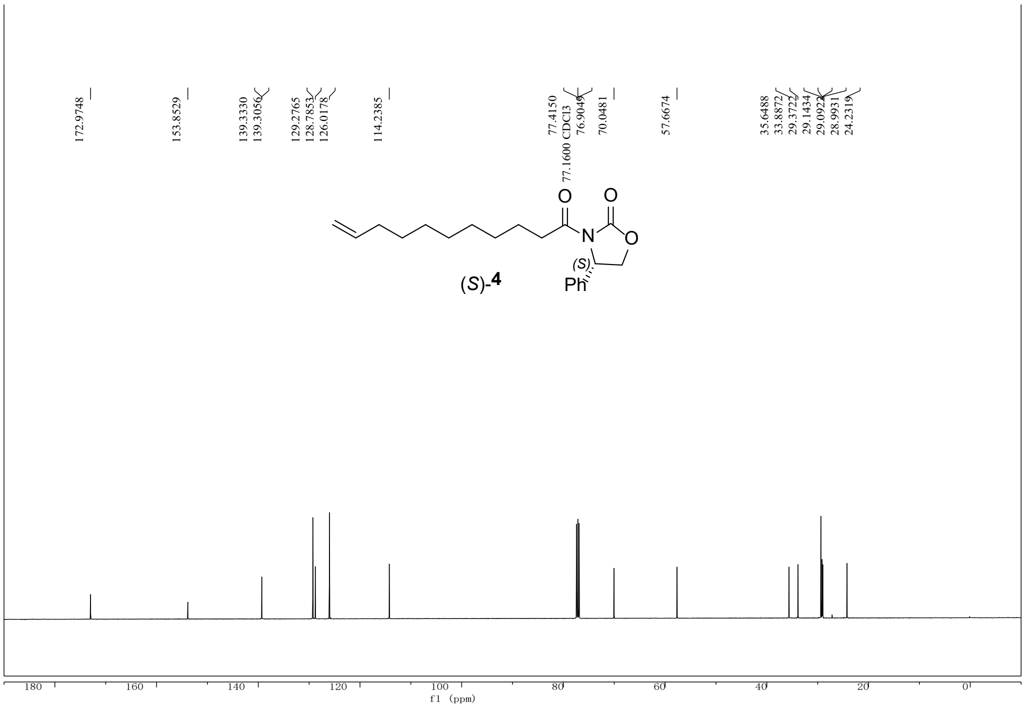

Figure S9. ^{1}H NMR spectrum of (*S*)-3-((*S*)-2-methylundec-10-enoyl)-4-phenyloxazolidin-2-one((*S*,*S*)-**5**) (500 MHz, $CDCl_3$).

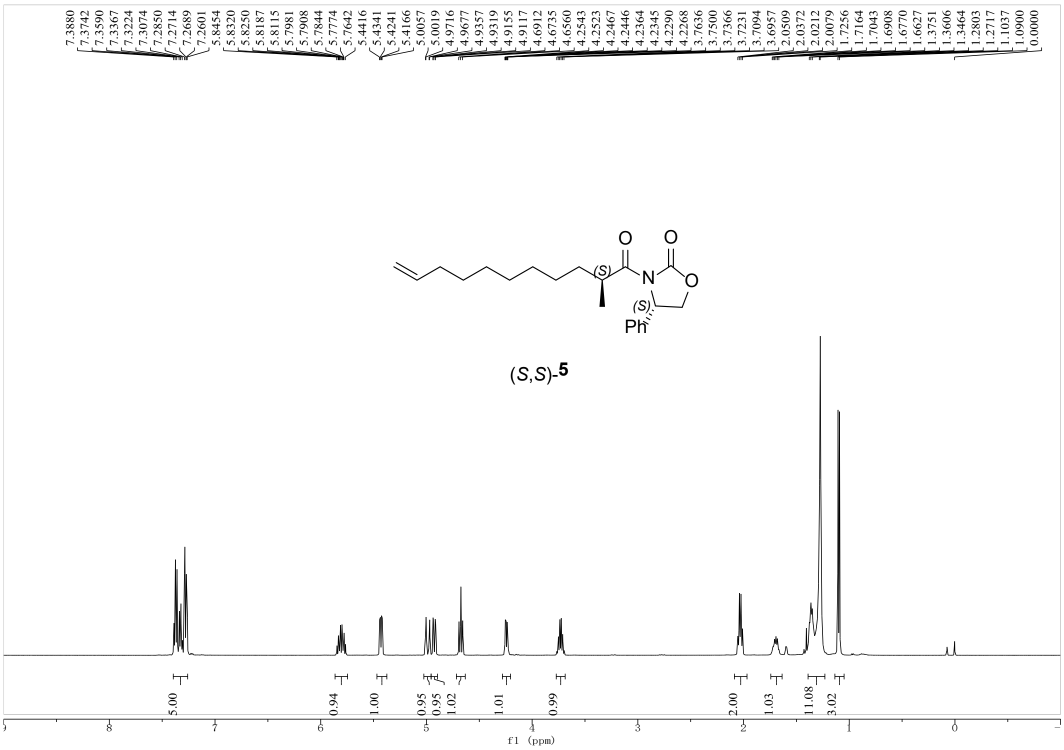

Figure S10. ^{13}C NMR spectrum of (*S*)-3-((*S*)-2-methylundec-10-enoyl)-4-phenyloxazolidin-2-one((*S*,*S*)-**5**) (126 MHz, $CDCl_3$).

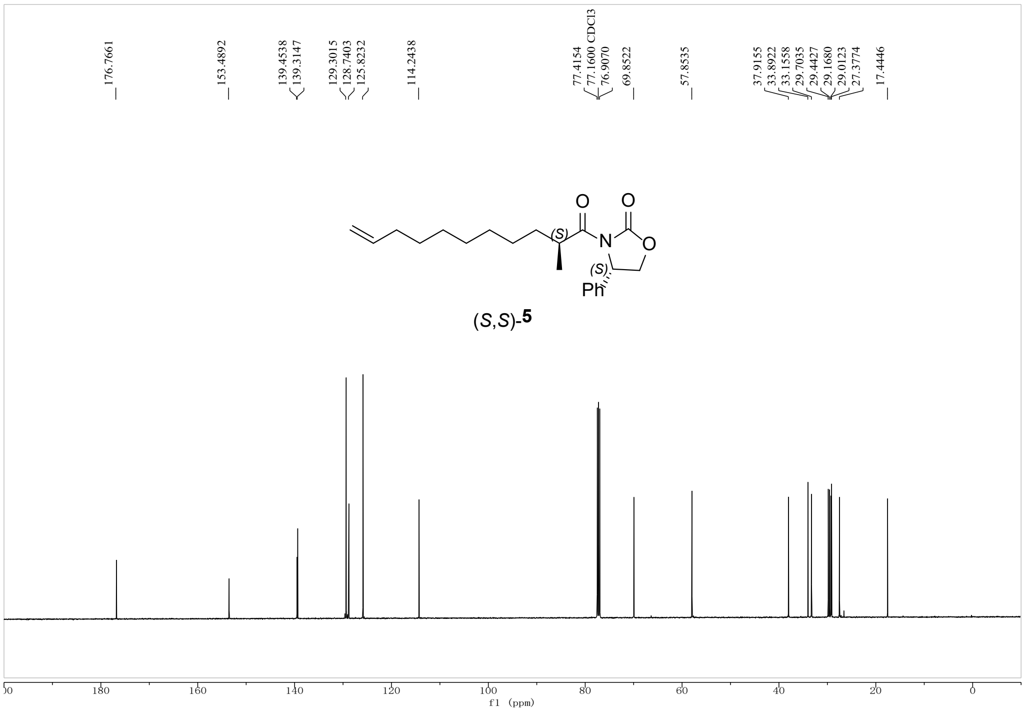

Figure S11. ^{1}H NMR spectrum of (*S*)-2-methylundec-10-en-1-ol ((*S*)-**6**) (500 MHz, $CDCl_3$).

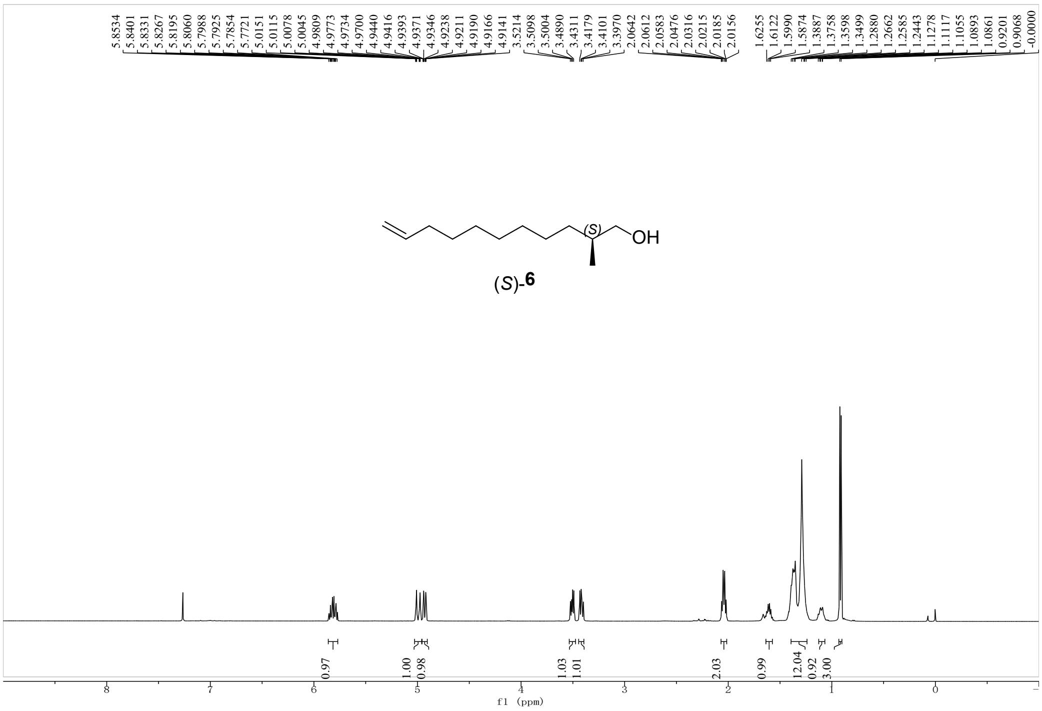

Figure S12. ^{13}C NMR spectrum of (*S*)-2-methylundec-10-en-1-ol ((*S*)-**6**) (126 MHz, $CDCl_3$).

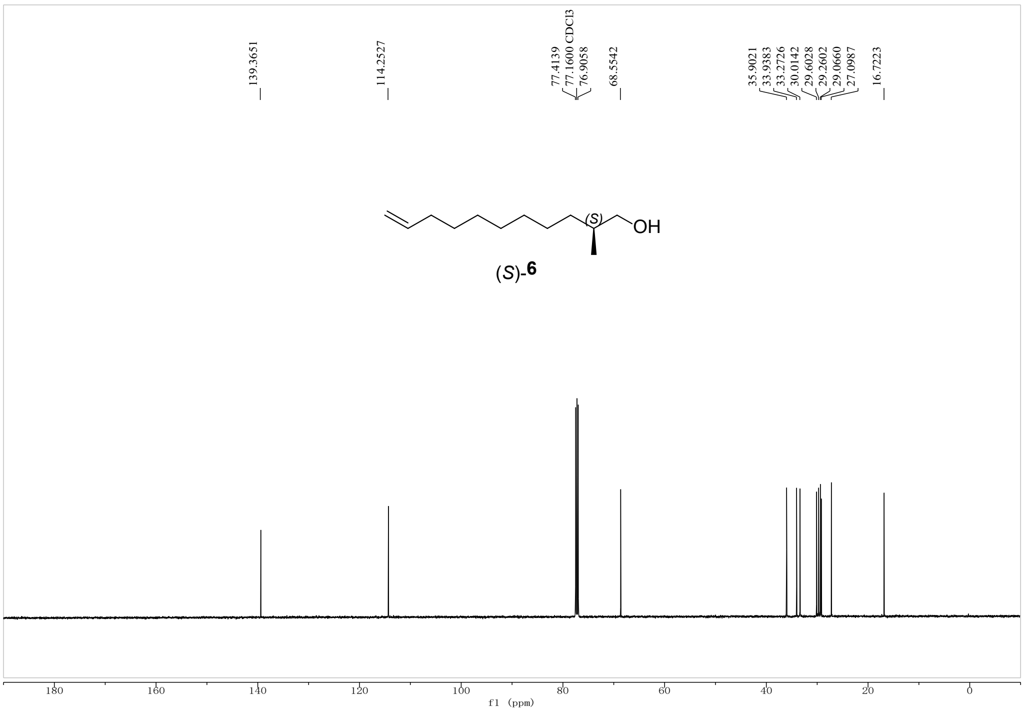

Figure S13. ^{1}H NMR spectrum of (*R*)-2-methylundec-10-en-1-yl 4-methylbenzenesulfonate ((*R*)-**7**) (500 MHz, $CDCl_3$).

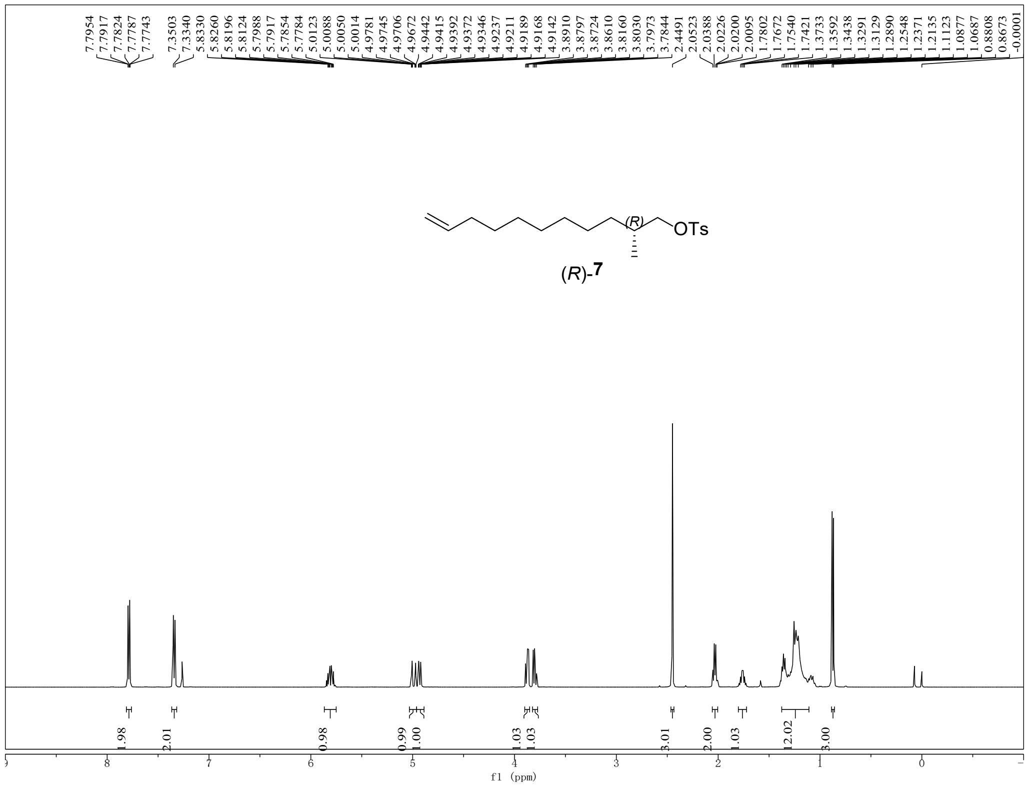

Figure S14. ^{13}C NMR spectrum of (*R*)-2-methylundec-10-en-1-yl 4-methylbenzenesulfonate ((*R*)-**7**) (126 MHz, C DCl3).

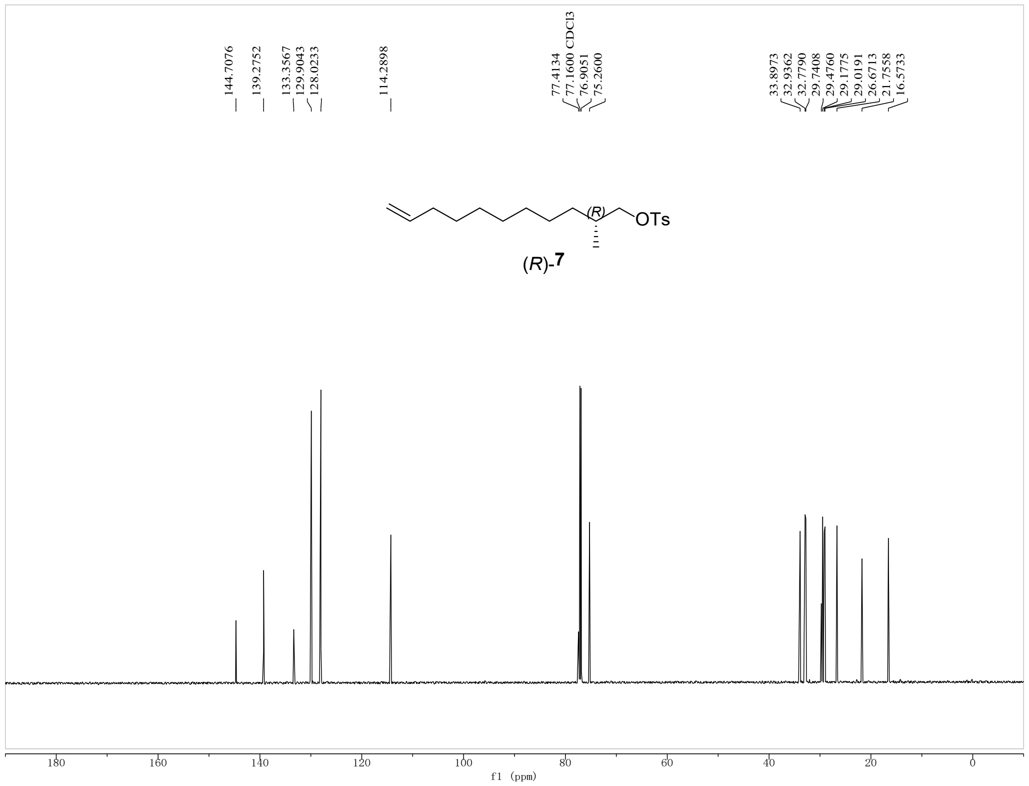

Figure S15. ^{1}H NMR spectrum of (*R*)-10,14-dimethylpentadec-1-ene ((*R*)-**9**) (500 MHz, $CDCl_3$).

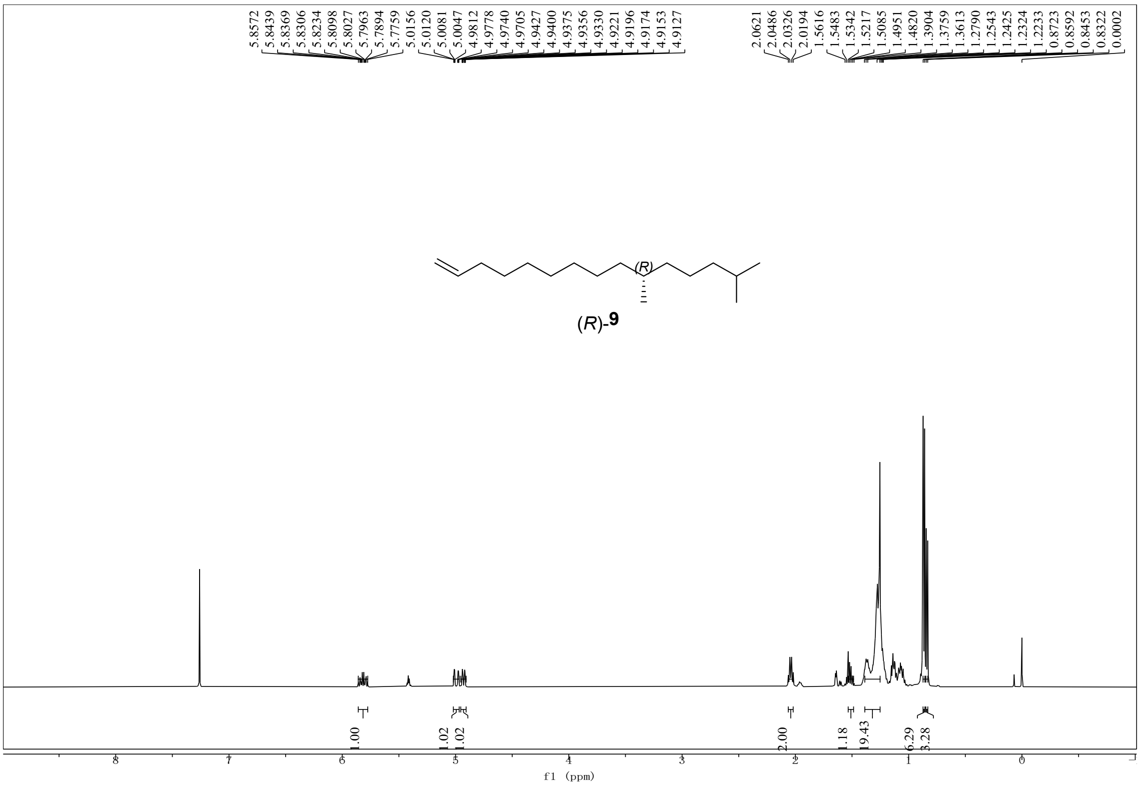

Figure S16. ^{13}C NMR spectrum of (*R*)-10,14-dimethylpentadec-1-ene ((*R*)-**9**) (126 MHz, $CDCl_3$).

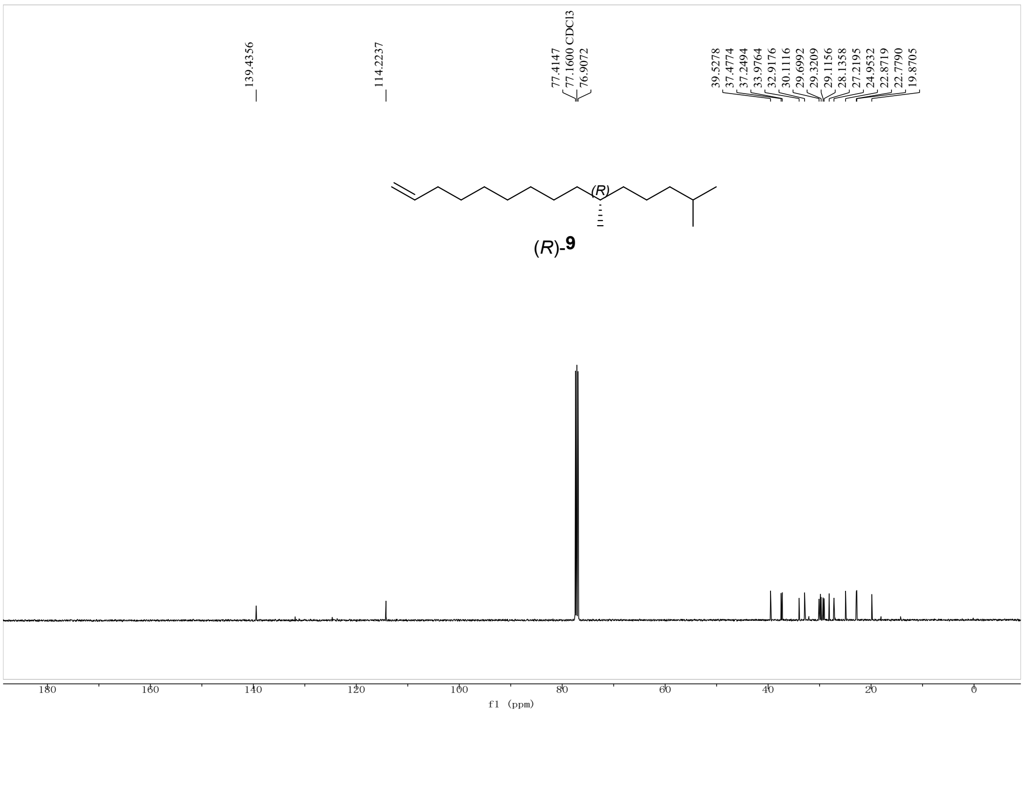

Figure S17. ^{1}H NMR spectrum of (*R*)-10,14-dimethylpentadecan-1-ol((*R*)-**10**) (500 MHz, $CDCl_3$).

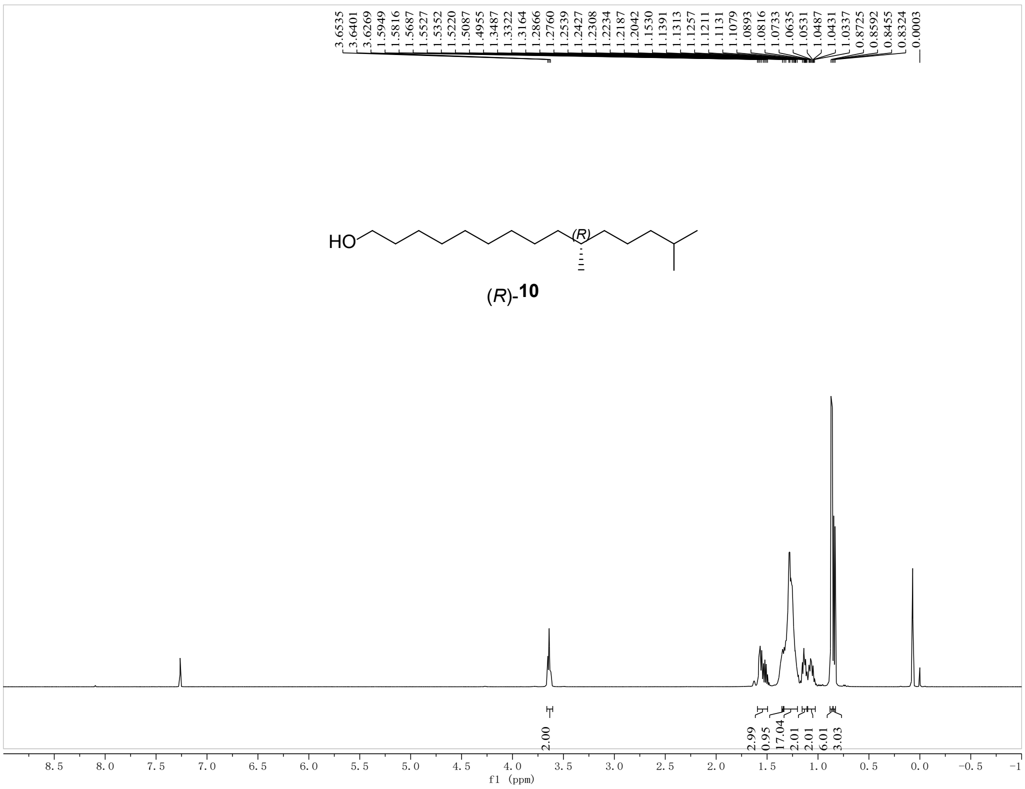

Figure S18. ^{13}C NMR spectrum of (*R*)-10,14-dimethylpentadecan-1-ol ((*R*)-**10**) (126 MHz, $CDCl_3$).

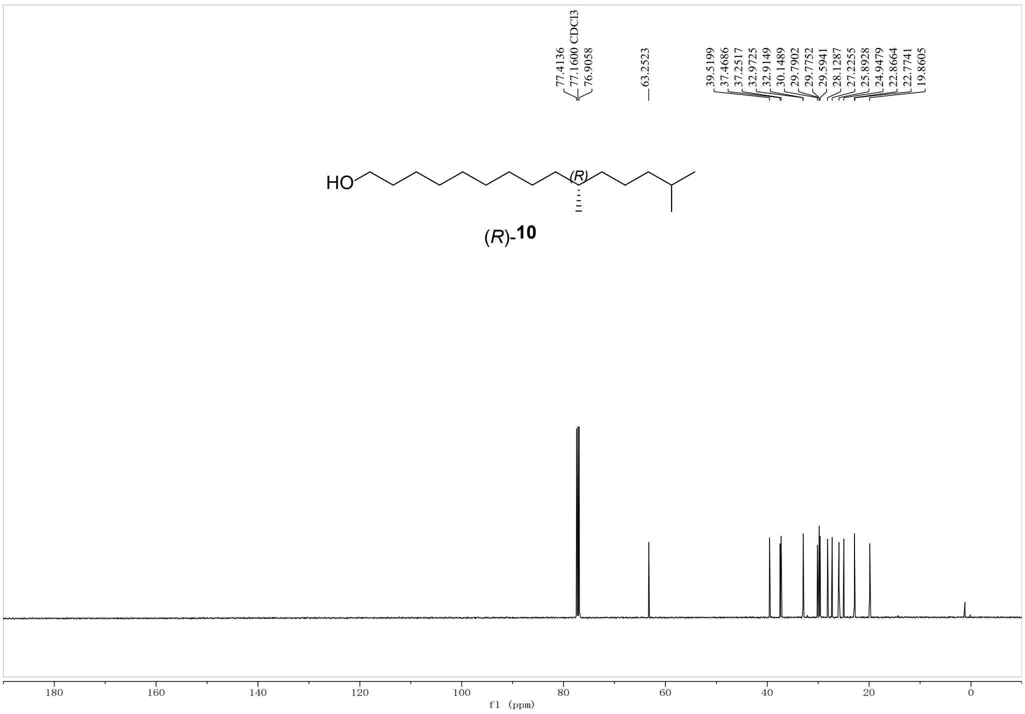

Figure S19. ^{1}H NMR spectrum of (*S*)-2-methylundec-10-en-1-yl 4-methylbenzenesulfonate ((*S*)-**7**) (500 MHz, $CDCl_3$).

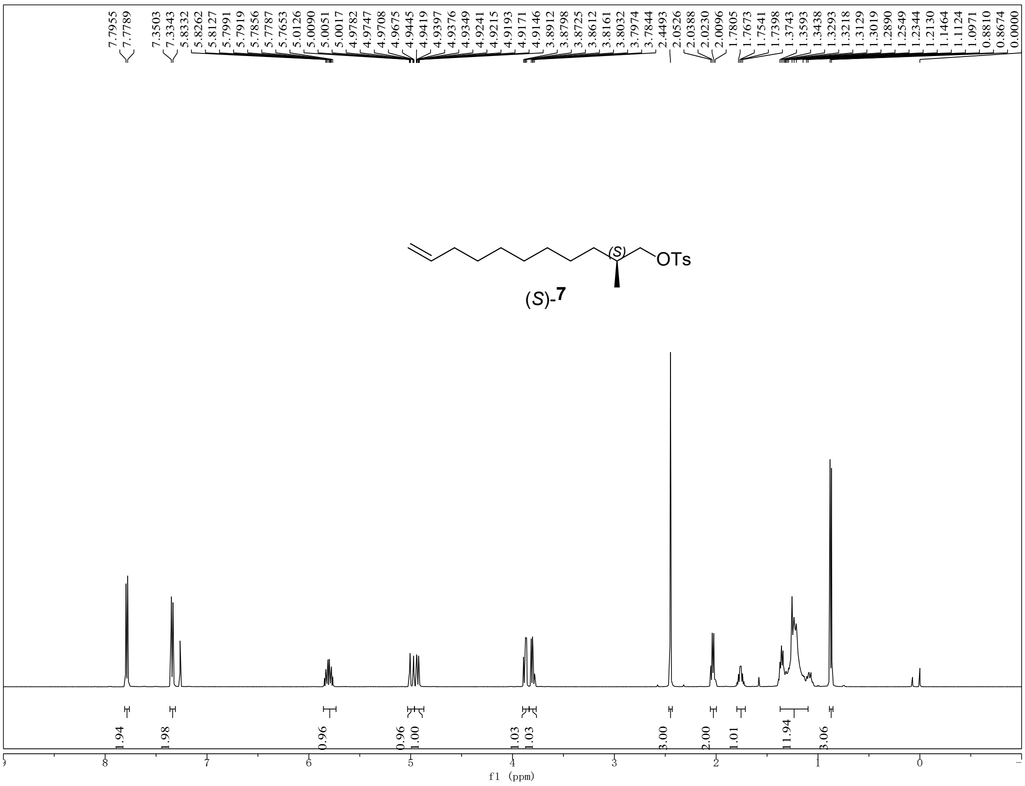

Figure S20. ^{13}C NMR spectrum of (*S*)-10,14-dimethylpentadec-1-ene ((*S*)-**7**) (126 MHz, $CDCl_3$).

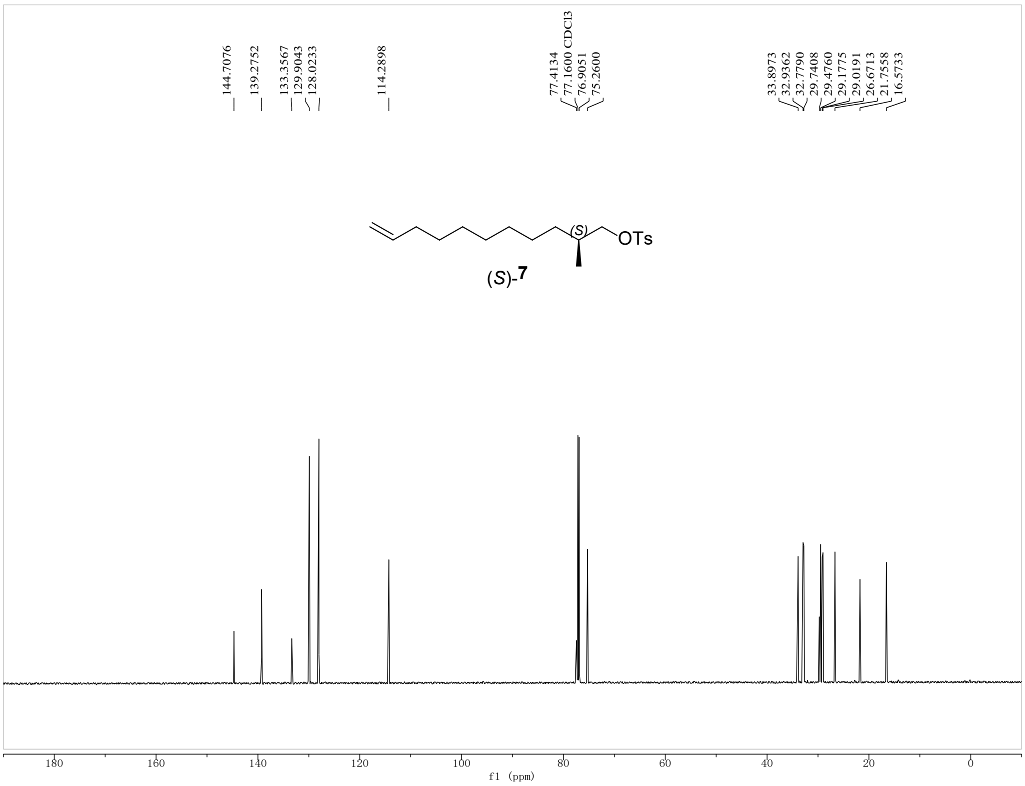

Figure S21. ^{1}H NMR spectrum of (*S*)-10,14-dimethylpentadec-1-ene ((*S*)-**9**) (500 MHz, $CDCl_3$).

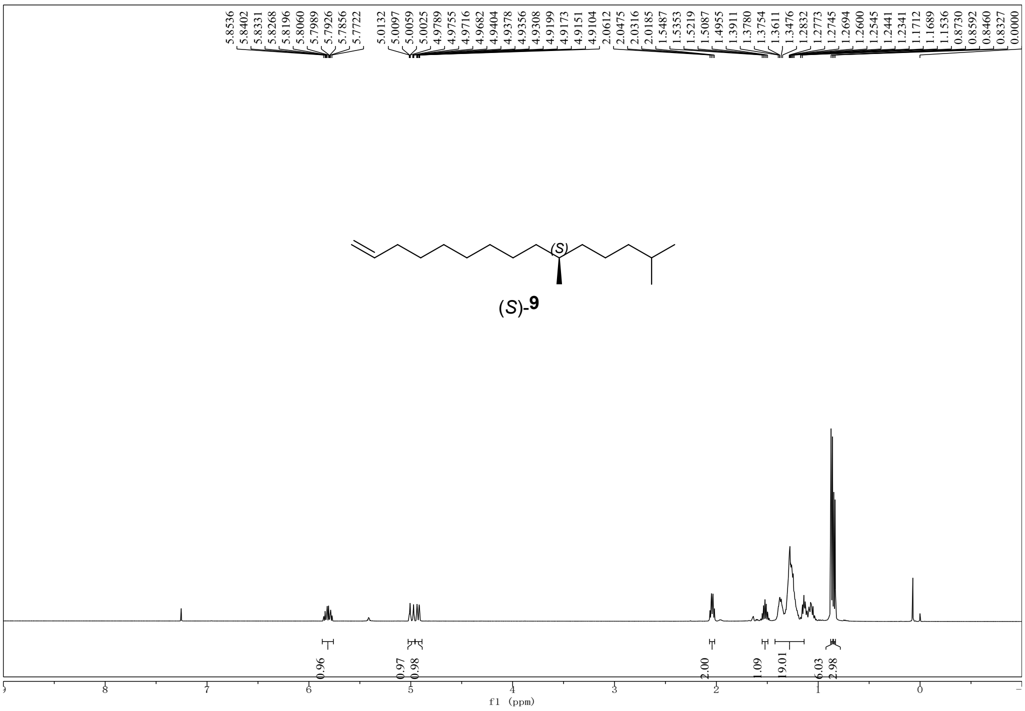

Figure S22. ^{13}C NMR spectrum of (*S*)-10,14-dimethylpentadec-1-ene ((*S*)-**9**) (126 MHz, $CDCl_3$).

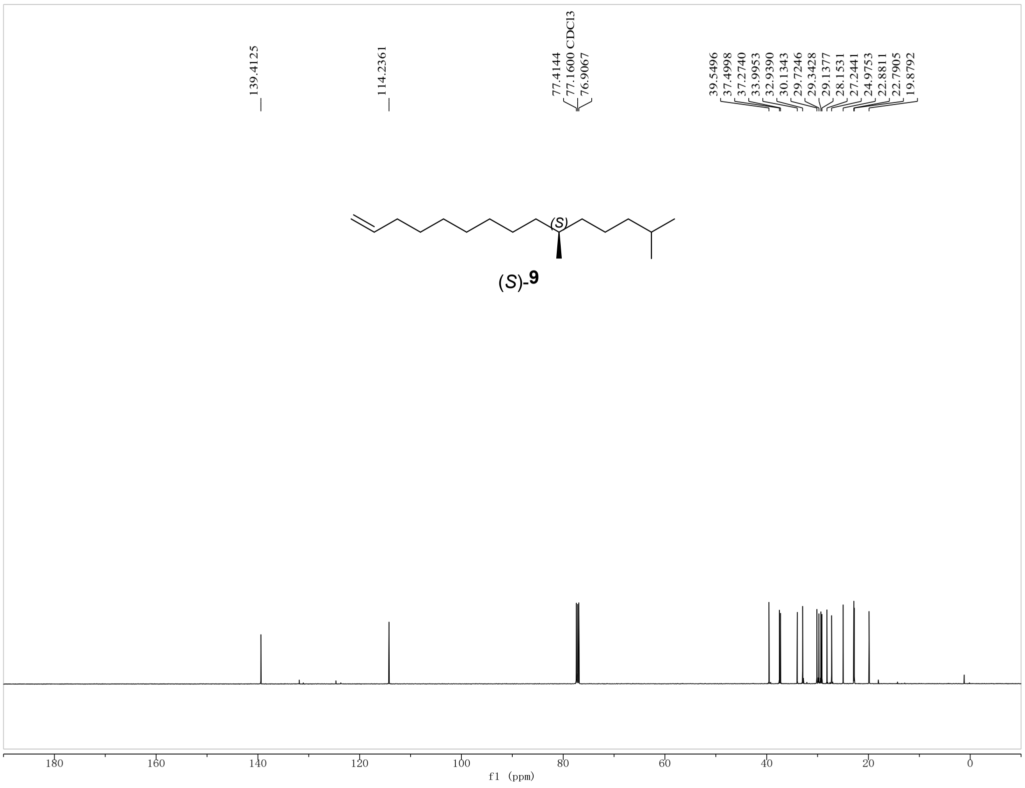

Figure S23. ^{1}H NMR spectrum of (*S*)-10,14-dimethylpentadecan-1-ol ((*S*)-**10**) (500 MHz, $CDCl_3$).

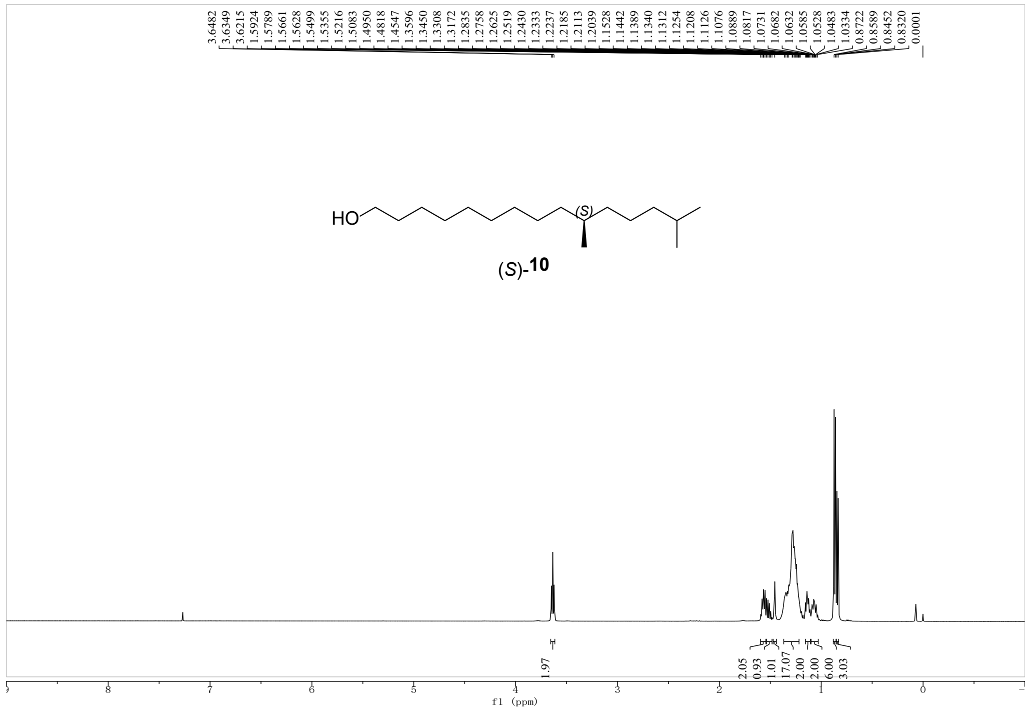

Figure S24. ^{13}C NMR spectrum of (*S*)-10,14-dimethylpentadecan-1-ol ((*S*)-**10**) (126 MHz, $CDCl_3$).

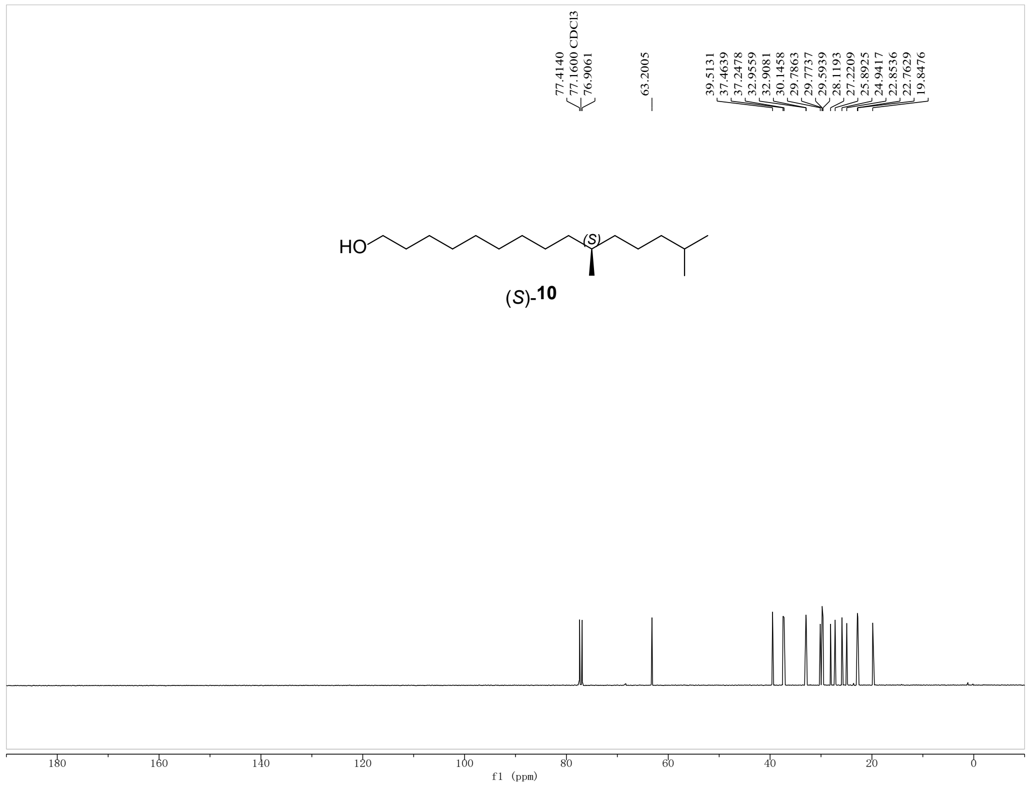

Figure S25. ^{1}H NMR spectrum of (*R*)-10,14-dimethylpentadecyl isobutyrate ((*R*)-**1**) (500 MHz, $CDCl_3$).

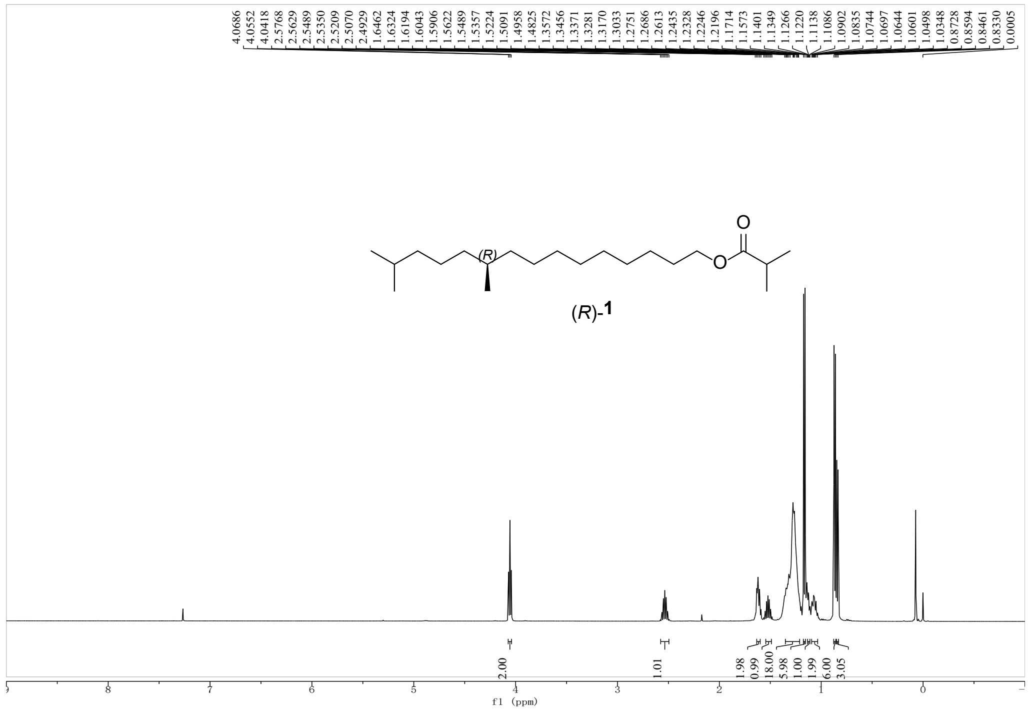

Figure S26. ^{13}C NMR spectrum of (*R*)-10,14-dimethylpentadecyl isobutyrate ((*R*)-**1**) (126 MHz, $CDCl_3$).

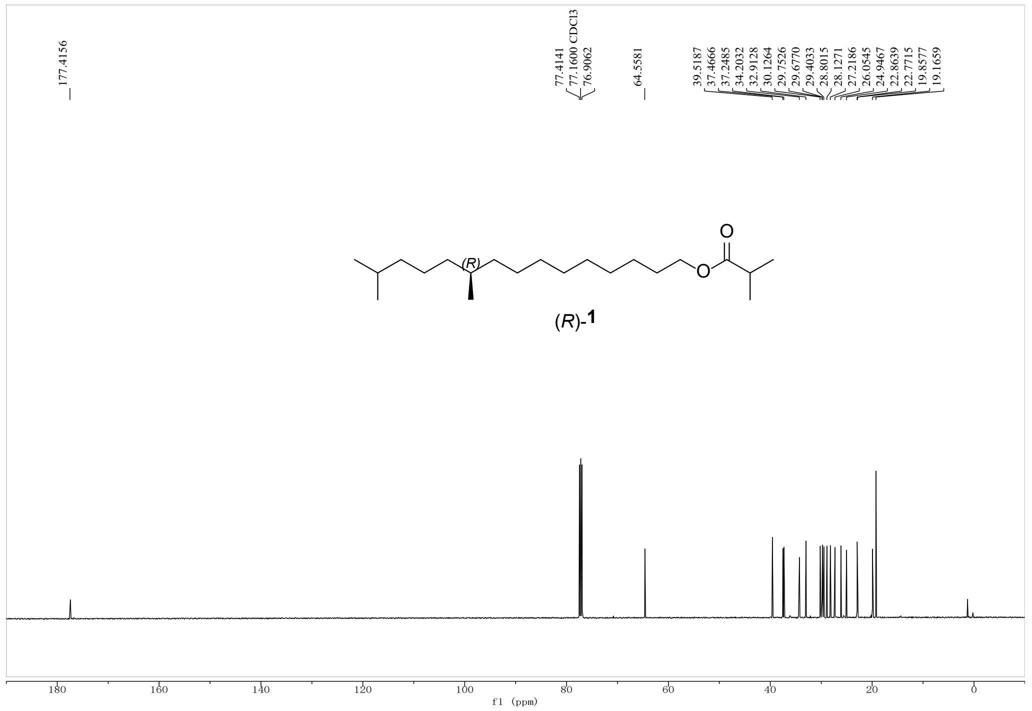

Figure S27. ^{1}H NMR spectrum of (*S*)-10,14-dimethylpentadecyl isobutyrate ((*S*)-**1**) (500 MHz, $CDCl_3$).

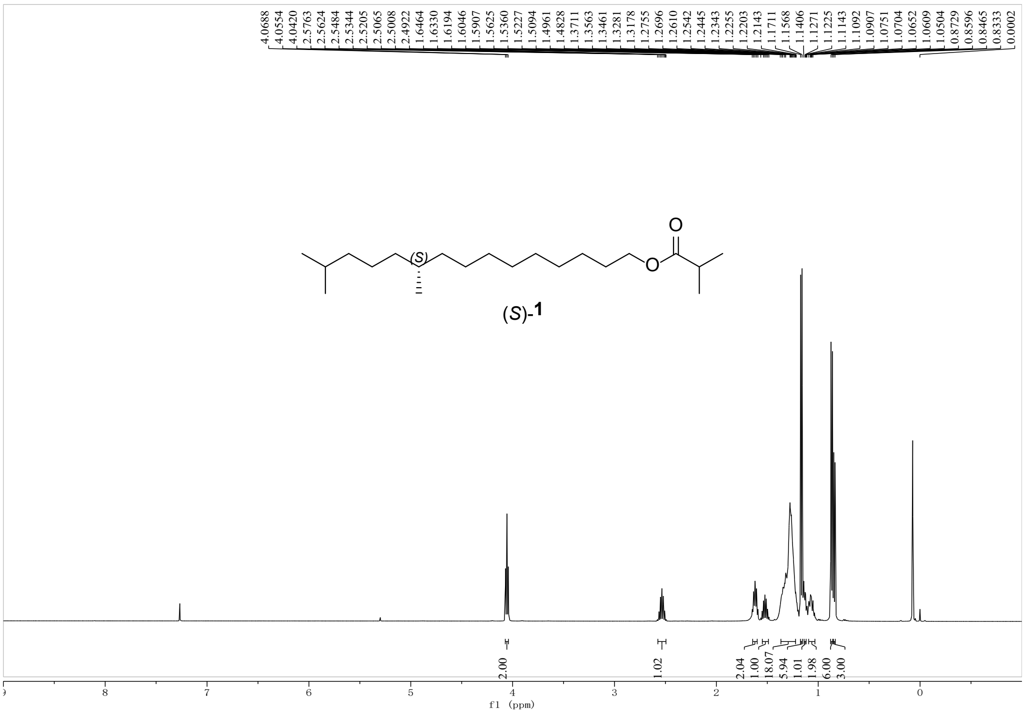

Figure S28. ^{13}C NMR spectrum of (*S*)-10,14-dimethylpentadecyl isobutyrate ((*S*)-**1**) (126 MHz, $CDCl_3$).

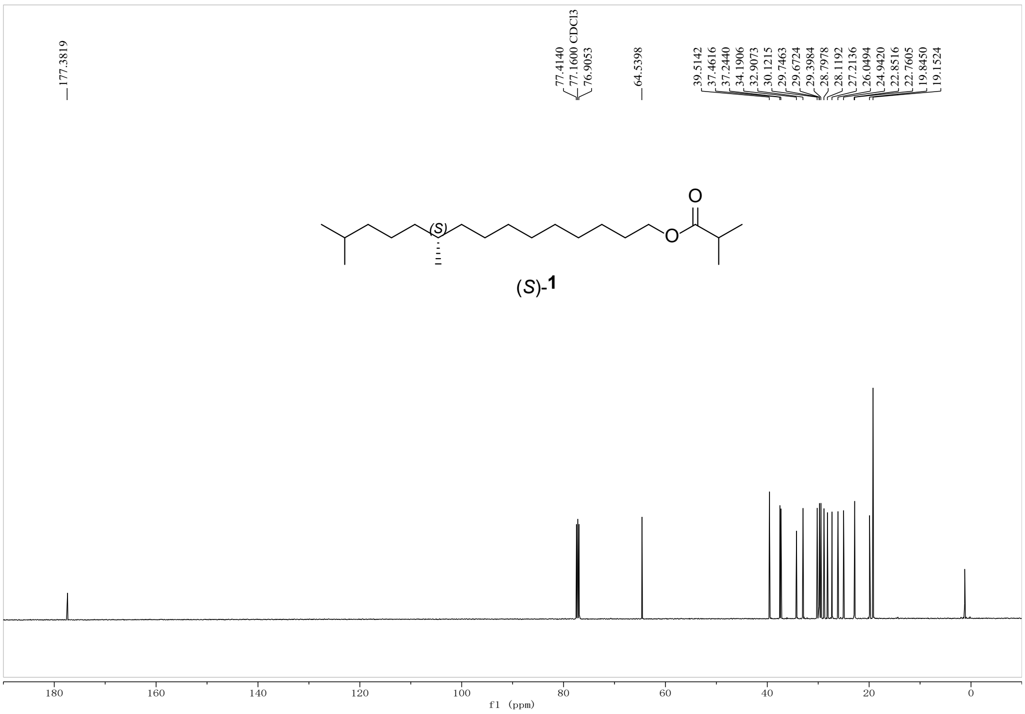

Figure S29. ^{1}H NMR spectrum of 3-(undec-10-enoyl)oxazolidin-2-one (**13**) (500 MHz, $CDCl_3$).

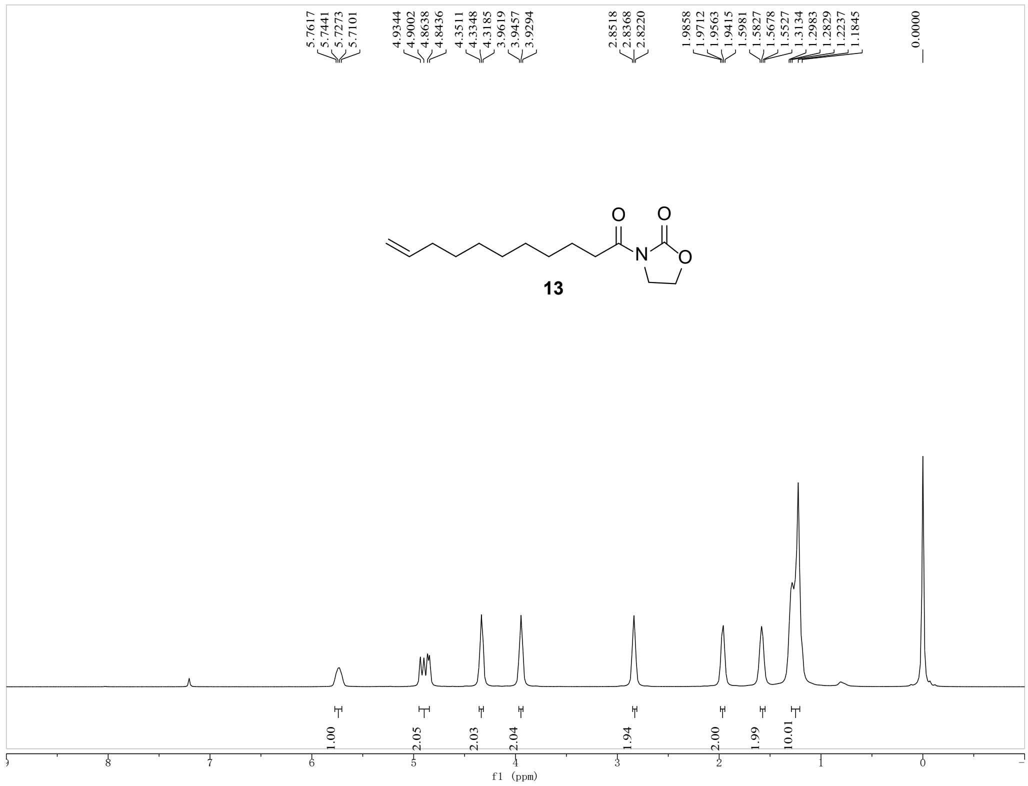

Figure S30. ^{13}C NMR spectrum of 3-(undec-10-enoyl)oxazolidin-2-one (**13**) (126 MHz, $CDCl_3$).

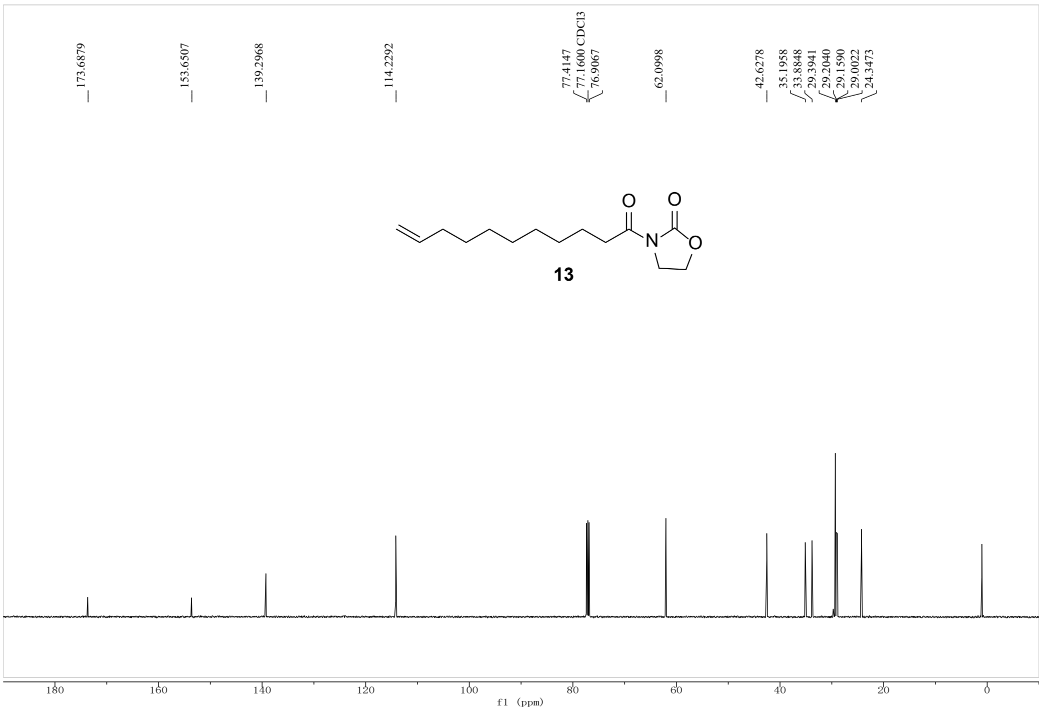

Figure S31. ^{1}H NMR spectrum of 3-(2-methylundec-10-enoyl)oxazolidin-2-one (**14**) (500 MHz, $CDCl_3$).

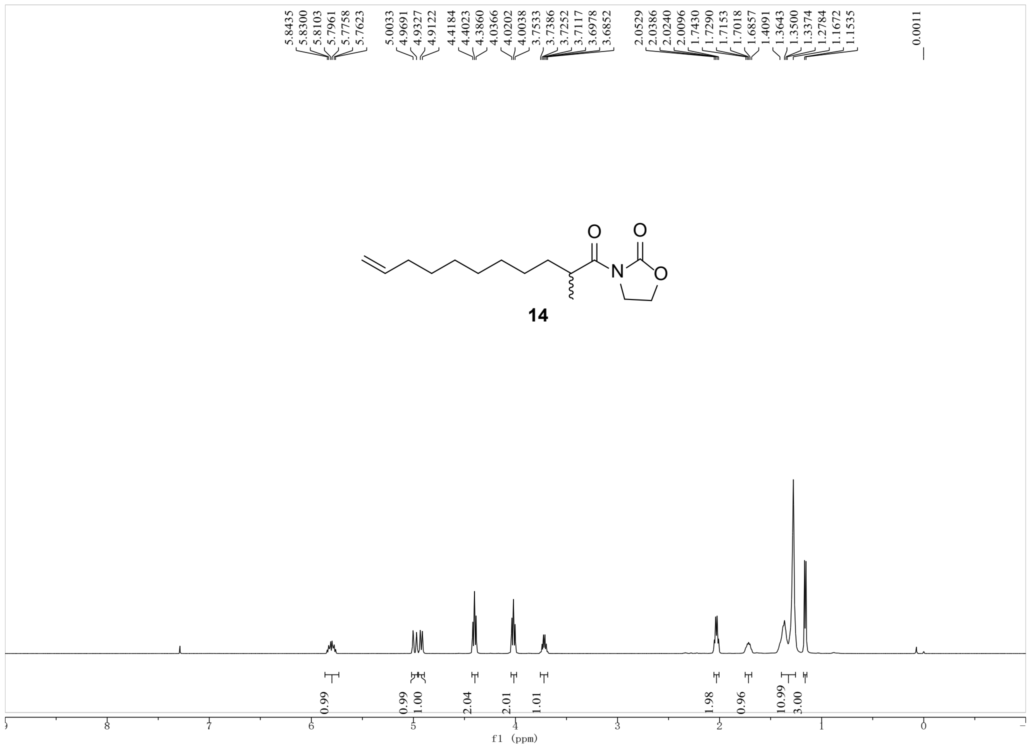

Figure S32. ^{13}C NMR spectrum of pentacosanal 3-(2-methylundec-10-enoyl)oxazolidin-2-one (**14**) (126 MHz, $CDCl_3$).

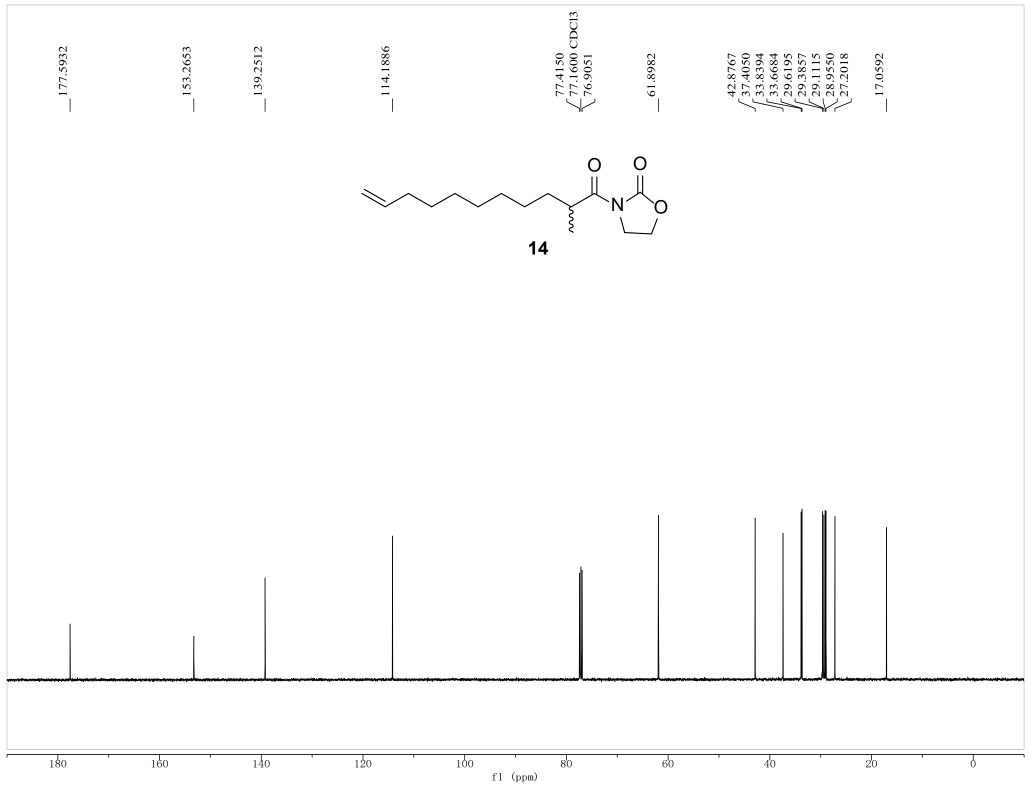

Figure S33. ^{1}H NMR spectrum of 2-methylundec-10-en-1-ol (*rac*-**6**) (500 MHz, $CDCl_3$).

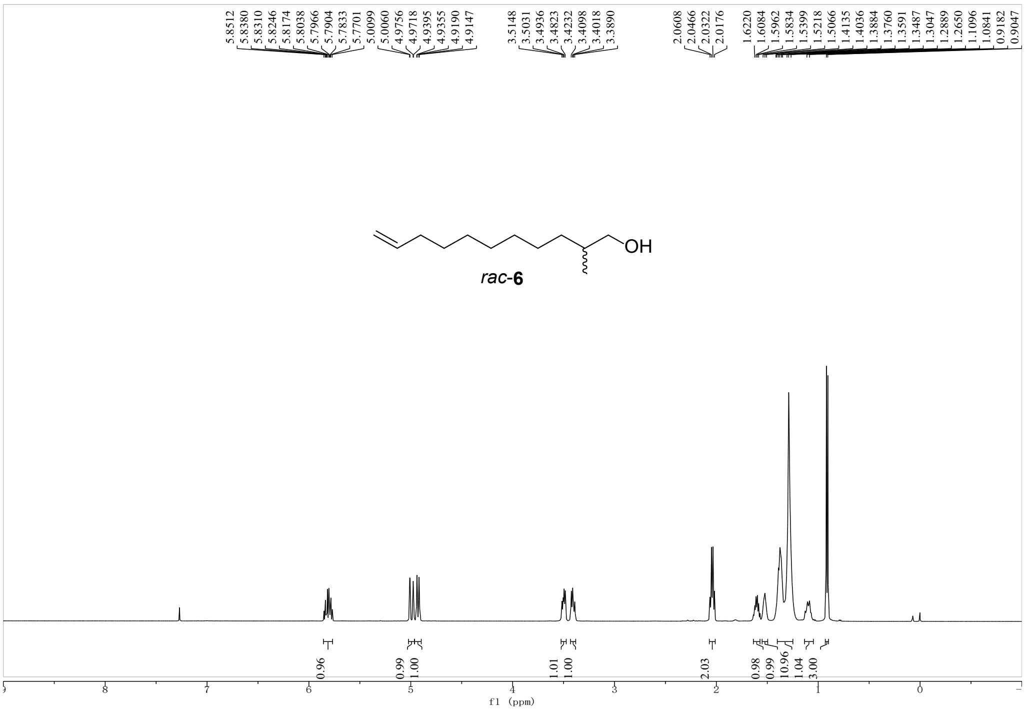

Figure S34. ^{13}C NMR spectrum of 2-methylundec-10-en-1-ol (*rac*-**6**) (126 MHz, $CDCl_3$).

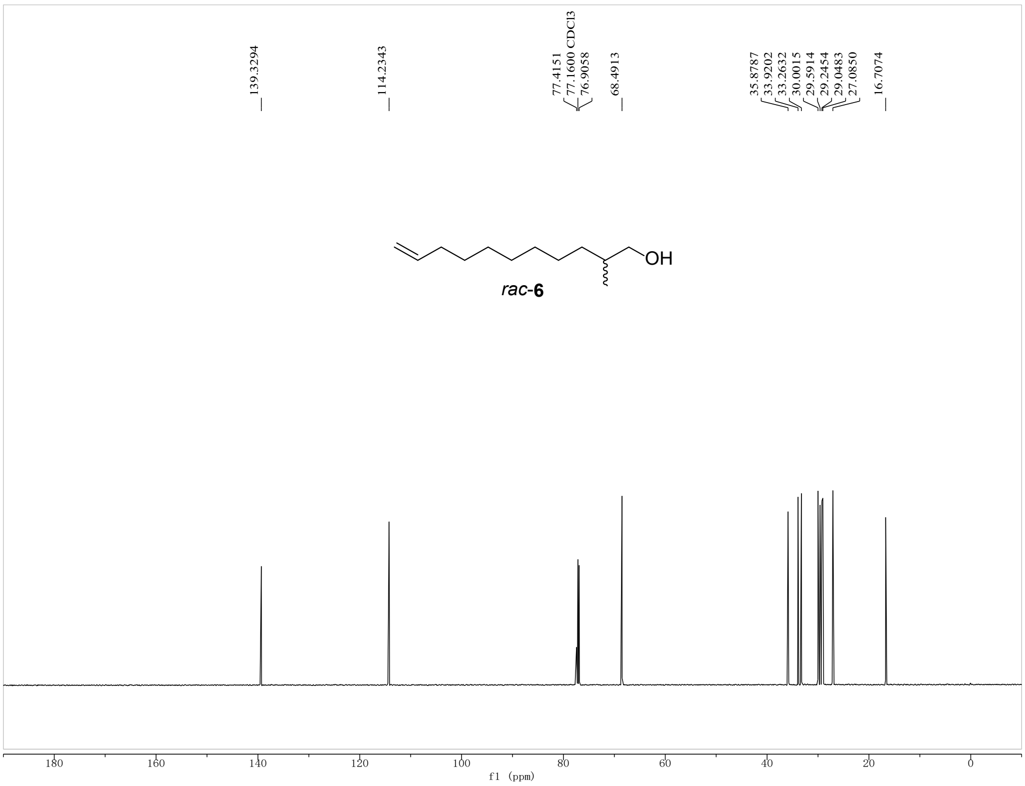

Figure S35. ^{1}H NMR spectrum of 2-methylundec-10-en-1-yl (*S*)-3,3,3-trifluoro-2-methoxy-2-phenylpropanoate (**16**) (500 MHz, $CDCl_3$).

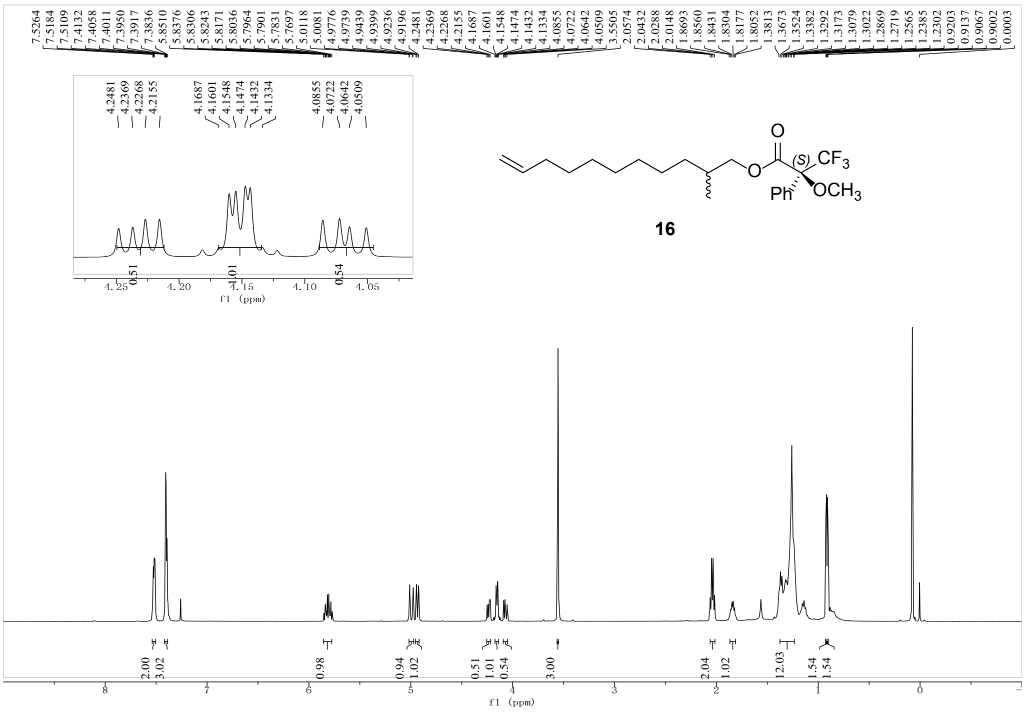

Figure S36. ^{13}C NMR spectrum of 2-methylundec-10-en-1-yl (*S*)-3,3,3-trifluoro-2-methoxy-2-phenylpropanoate (**16**) (126 MHz, $CDCl_3$).

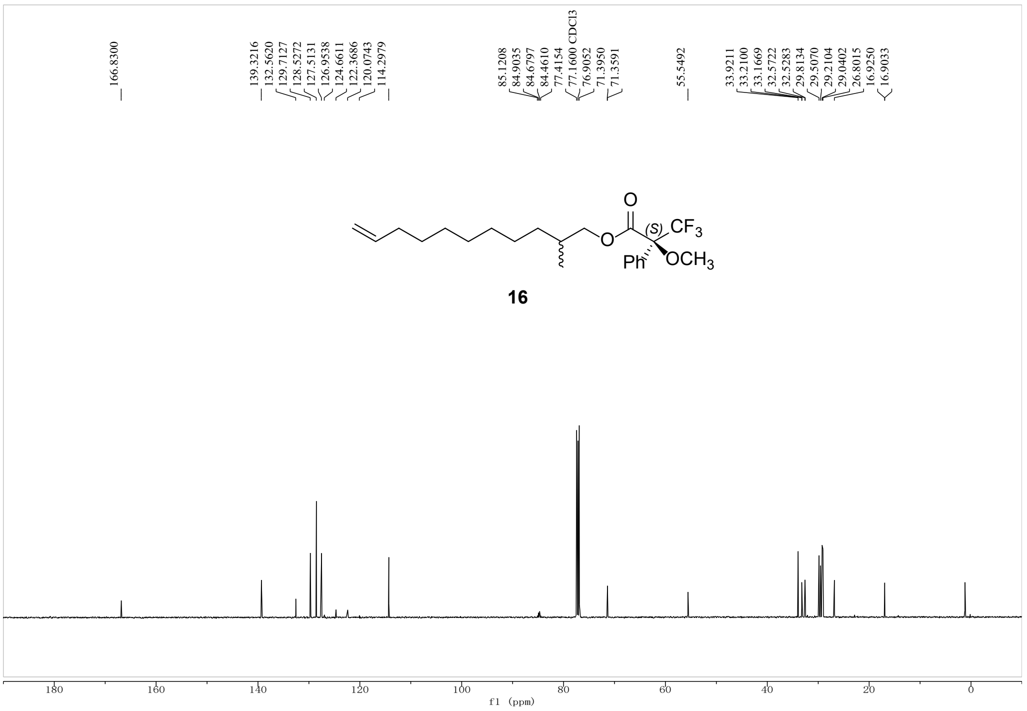

Figure S37. ^{19}F NMR spectrum of 2-methylundec-10-en-1-yl (*S*)-3,3,3-trifluoro-2-methoxy-2-phenylpropanoate (**16**) (471 MHz, $CDCl_3$).

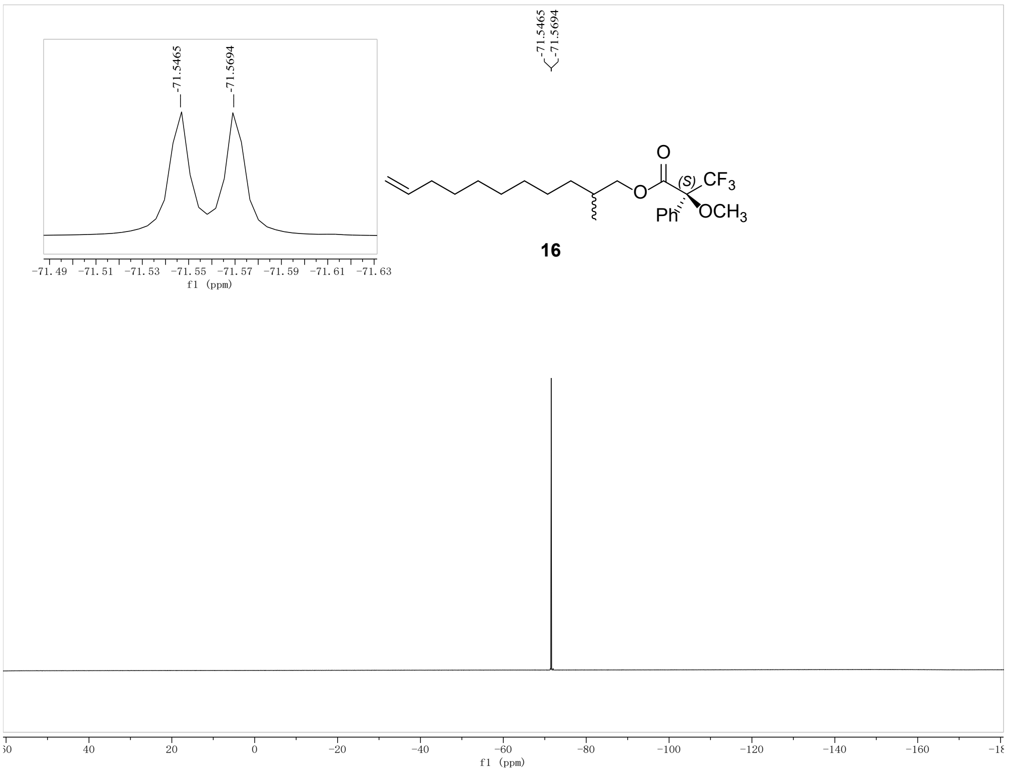

Figure S38. ^{1}H NMR spectrum of (*S*)-2-methylundec-10-en-1-yl (*S*)-3,3,3-trifluoro-2-methoxy-2-phenylpropanoate (**17**) (500 MHz, $CDCl_3$).

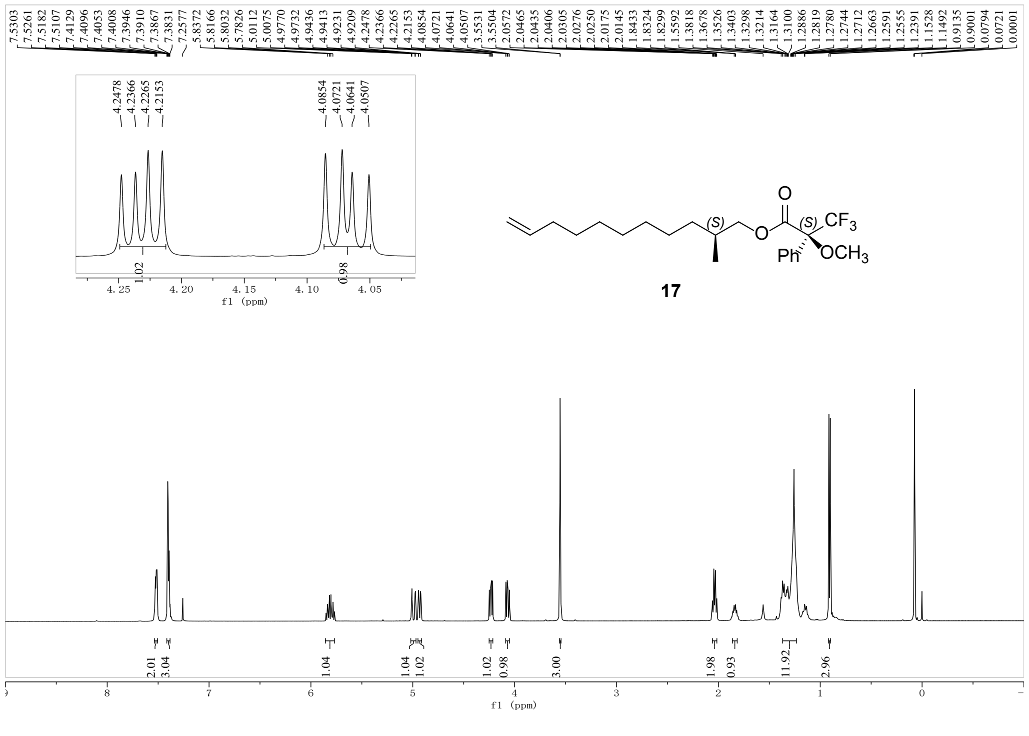

Figure S39. ^{13}C NMR spectrum of (*S*)-2-methylundec-10-en-1-yl (*S*)-3,3,3-trifluoro-2-methoxy-2-phenylpropanoate (**17**) (126 MHz, $CDCl_3$).

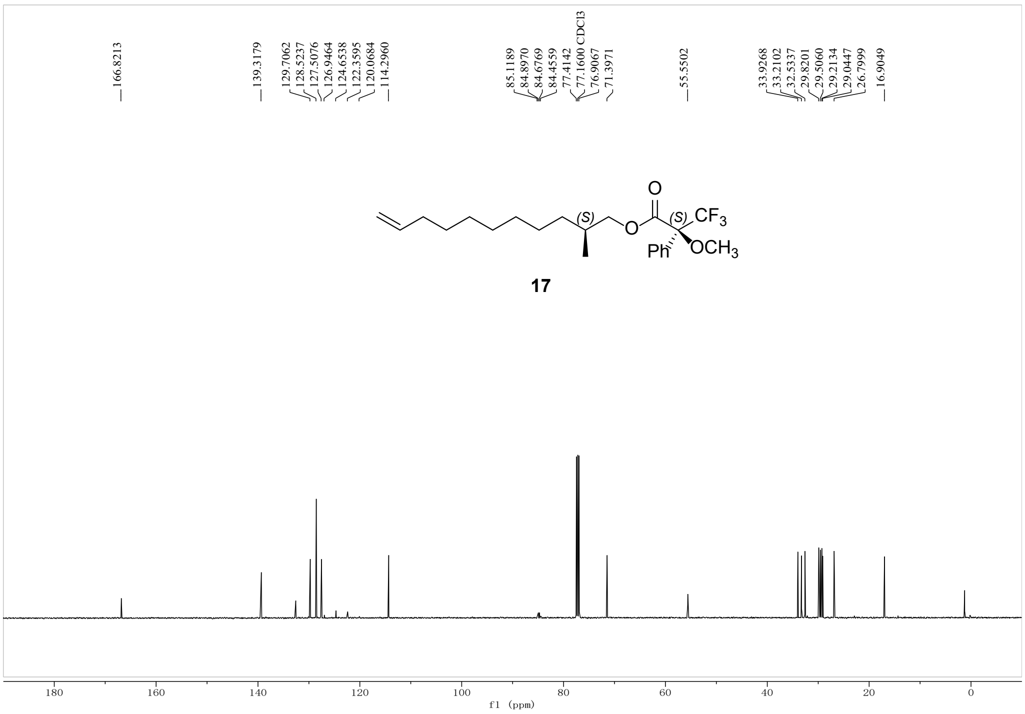

Figure S40. ^{19}F NMR spectrum of (*S*)-2-methylundec-10-en-1-yl (*S*)-3,3,3-trifluoro-2-methoxy-2-phenylpropanoate (**17**) (471 MHz, $CDCl_3$).

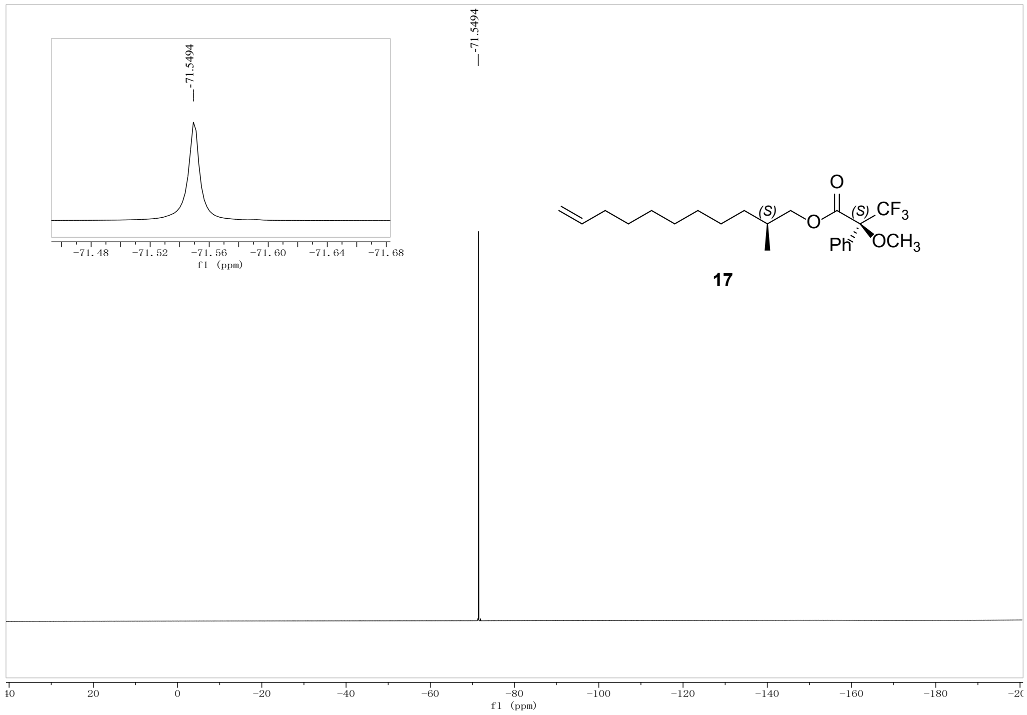

Figure S41. ^{1}H NMR spectrum of (*R*)-2-methylundec-10-en-1-yl (*S*)-3,3,3-trifluoro-2-methoxy-2-phenylpropanoate (**18**) (500 MHz, $CDCl_3$).

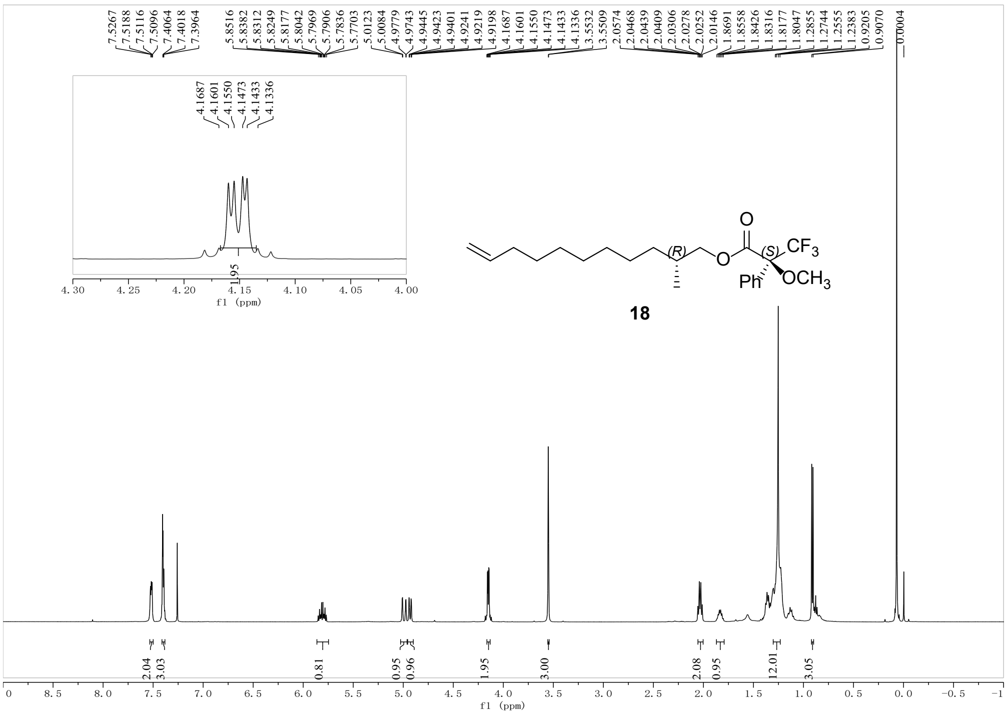

Figure S42. ^{13}C NMR spectrum of (*R*)-2-methylundec-10-en-1-yl (*S*)-3,3,3-trifluoro-2-methoxy-2-phenylpropanoate (**18**) (126 MHz, $CDCl_3$).

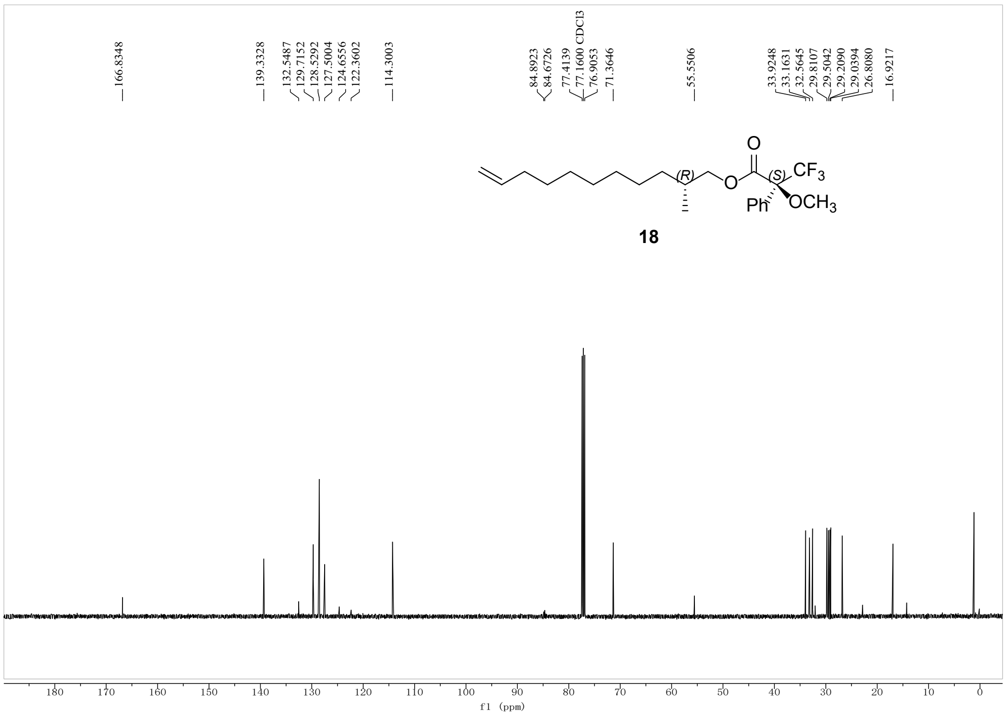

Figure S43. ^{19}F NMR spectrum of (*R*)-2-methylundec-10-en-1-yl (*S*)-3,3,3-trifluoro-2-methoxy-2-phenylpropanoate (**18**) (471 MHz, $CDCl_3$).

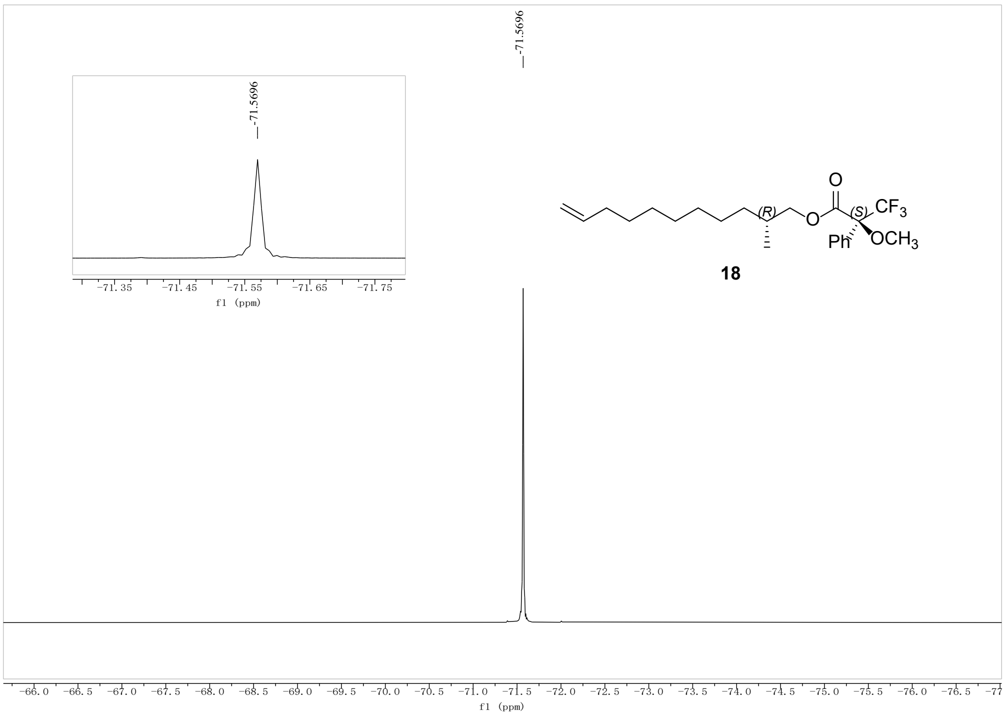

4. References

1. Peddie, V.; Butcher, R. J.; Robinson, W. T.; Wilce, M. C. J.; Traore, D. A. K.; Abell, A. D. Synthesis and conformation of fluorinated β-peptidic compounds. *Chem. Eur. J.* **2012**, *18*, 6655-6662.
2. Fuwa, H.; Nakajima, M.; Shi, J.; Takeda, Y.; Saito, T.; Sasaki, M. A convergent synthesis of the C1-C16 segment of goniodomin a via palladium-catalyzed organostannane-thioester coupling. *Org. Lett.* **2011**, *13*, 1106-1109.
3. Evans, D. A.; Ennis, M. D.; Mathre, D. J. Asymmetric alkylation reactions of chiral imide enolates. A practical approach to the enantioselective synthesis of α-substituted carboxylic acid derivatives. *J. Am. Chem. Soc.* **1982**, *104*, 1737-1739.
4. Wu, J.; Lin, C.; Liu, D.; Bian, Q.; Wang, M.; Zhong, J. Total synthesis of (6*R*,12*R*)-6,12-dimethylpentadecan-2-one, the sex pheromone of *Diabrotica balteata* Leconte. *Chirality* **2024**, *36*, e23658.
5. Wu, Y.; Shen, X.; Tang, C.-J.; Chen, Z.-L.; Hu, Q.; Shi, W. Synthesis of natural fragrant molecules cis-3-methyl-4-decanolide and aerangis lactone. General enantioselective routes to β,γ-cis-disubstituted γ-lactones and γ,δ-cis-disubstituted δ-lactones. *J. Org. Chem.* **2002**, *67*, 3802-3810.
6. Tsuda, M.; Toriyabe, Y.; Endo, T.; Kobayashi, J. i. Application of modified Mosher's method for primary alcohols with a methyl group at C2 position. *Chem. Pharm. Bull.* **2003**, *51*, 448-451.
7. Dale, J. A.; Mosher, H. S. Nuclear magnetic resonance enantiomer regents. Configurational

correlations via nuclear magnetic resonance chemical shifts of diastereomeric mandelate, O-methylmandelate, and α-methoxy-α-trifluoromethylphenylacetate (MTPA) esters. *J. Am. Chem. Soc.* **1973**, *95*, 512-519.
